# Antiproliferative Activity of *Cyanophora paradoxa* Pigments in Melanoma, Breast and Lung Cancer Cells

**DOI:** 10.3390/md11114390

**Published:** 2013-11-01

**Authors:** Paul-Hubert Baudelet, Anne-Laure Gagez, Jean-Baptiste Bérard, Camille Juin, Nicolas Bridiau, Raymond Kaas, Valérie Thiéry, Jean-Paul Cadoret, Laurent Picot

**Affiliations:** 1 University of La Rochelle, UMRi CNRS 7266 LIENSs, F-17042, La Rochelle, France; E-Mails: paulhubertbaudelet@gmail.com (P.-H.B.); anne-laure.gagez@univ-lr.fr (A.-L.G.); camille.juin@univ-lr.fr (C.J.); nicolas.bridiau@univ-lr.fr (N.B.); valerie.thiery@univ-lr.fr (V.T.); 2 University of La Rochelle, UMRi CNRS 7266 LIENSs, Platform for the High Resolution Analysis of Biomolecules, F-17071, La Rochelle cedex 9, France; 3 IFREMER, Laboratory PBA, IFREMER Centre Nantes, F-44311, Nantes, France; E-Mails: jean.baptiste.berard@ifremer.fr (J.-B.B.); raymond.kaas@ifremer.fr (R.K.); jean.paul.cadoret@ifremer.fr (J.-P.C.)

**Keywords:** pigments, microalgae, *Cyanophora paradoxa*, melanoma, cancer, apoptosis, zeaxanthin, cryptoxanthin

## Abstract

The glaucophyte *Cyanophora paradoxa* (*Cp*) was chemically investigated to identify pigments efficiently inhibiting malignant melanoma, mammary carcinoma and lung adenocarcinoma cells growth. *Cp* water and ethanol extracts significantly inhibited the growth of the three cancer cell lines *in vitro*, at 100 µg·mL^−1^. Flash chromatography of the *Cp* ethanol extract, devoid of c-phycocyanin and allophycocyanin, enabled the collection of eight fractions, four of which strongly inhibited cancer cells growth at 100 µg·mL^−1^. Particularly, two fractions inhibited more than 90% of the melanoma cells growth, one inducing apoptosis in the three cancer cells lines. The detailed analysis of *Cp* pigment composition resulted in the discrimination of 17 molecules, ten of which were unequivocally identified by high resolution mass spectrometry. Pheophorbide a, β-cryptoxanthin and zeaxanthin were the three main pigments or derivatives responsible for the strong cytotoxicity of *Cp* fractions in cancer cells. These data point to *Cyanophora paradoxa* as a new microalgal source to purify potent anticancer pigments, and demonstrate for the first time the strong antiproliferative activity of zeaxanthin and β-cryptoxanthin in melanoma cells.

## 1. Introduction

Microalgae have a great potential for the production of bioactive molecules of pharmaceutical interest [1,2,3,4,5]. Particularly, microalgal pigments protect normal cells from genetic damages and exert antiproliferative, cytotoxic and pro-apoptotic activities in cancer cells, suggesting their possible use for cancer prevention or chemiotherapy [6,7,8]. The biological activity of microalgal pigments is explained by a variety of physicochemical and pharmacological actions [6]. Most exert antioxidant activities, protect cells from UV or ROS-induced DNA alterations and limit inflammation and mutagenesis. Some pigments cross the lipophilic membranes and may interact with membrane proteins involved in cancer cells multi-drug resistance, or apoptosis. Some also inhibit DNA-dependent DNA polymerases, alter the expression of cyclins and CDK, or interfere with major transduction pathways controlling cell survival and transcriptional activation of genes involved in apoptosis, anticancer drugs resistance or Gap junction intercellular communication. The anticancer activity of microalgal pigments may also be related to their angiostatic activity and to their ability to stimulate antitumoral immune responses. Finally, the relevance of microalgae pigments as non-apoptotic cell death inducers, antimetastatic, or inhibitors of cancer cells invasivity or motility, remains to be established. We recently demonstrated the antiproliferative activity of violaxanthin in breast cancer cells, using a bioguided purification strategy [7], and reviewed the anticancer activity of microalgal epoxycarotenoids [6]. We here demonstrate that pigments from the glaucocystophyte *Cyanophora paradoxa* may have interest as anticancer agents, particularly to cure malignant melanomas, which are frequent cancers with bad prognosis.

## 2. Results and Discussion

### 2.1. Antiproliferative Activity of *Cp* Extracts

Growth inhibition was determined after a 72 h treatment with *Cp* extracts 100 µg·mL^−1^, to discriminate antiproliferative extracts (Table 1).

**Table 1:** Growth inhibition (%) after a 72 h treatment with 100 µg·mL^−1^ *Cp* raw extracts. DCM: dichloromethane; EtOH: ethanol. *t* test (treated *vs*. control): * *p <* 0.05. ND: non determinable. Negative values indicate stimulation of cell growth.

	Cell line		
	A-549 Lung adenocarcinoma	MCF-7 Mammary carcinoma	A-2058 Malignant melanoma
DCM extract	−15.4 ± 2.7	−14.6 ± 1.8	53.3 ± 4.8 *
EtOH extract 1	26.2 ± 2.8	22.2 ± 2.2	61.8 ± 4.1 *
EtOH extract 2	23.3 ± 0.3	23.8 ± 0.2	26.6 ± 0.3 *
Water extract 1	ND	ND	ND
Water extract 2	8.6 ± 0.3	25.5 ± 0.4 *	19.4 ± 0.3

The DCM extract did not induce A-549 and MCF-7 growth inhibition, but significantly inhibited the A-2058 melanoma cell line. The ethanol extracts 1 and 2 induced a high growth inhibition in the three cancer cell lines, and ethanol extract 2 was selected for further purification of antiproliferative molecules as it was devoid of phycocyanin, removed in the water extract 2 during the polar cascade extraction. The significant growth inhibition of A-2058 cells by *Cp* extracts drew our attention as malignant melanoma cells are usually difficult to kill, and invasive melanoma have a very poor prognosis [9]. The A-2058 cell line was thus selected for the bio-guided purification of antiproliferative molecules from the ethanol extract 2. The water extract 1 was not studied as the mass recovered after rotavapor drying was negligible, and it was thus impossible to solubilize it in the cell culture medium to obtain a 100 µg·mL^−1^ concentration.

### 2.2. RP-HPLC Analysis, Fractionation of the Ethanol Extract, and Antiproliferative Activity of Flash Chromatography Fractions

**Figure 1:**
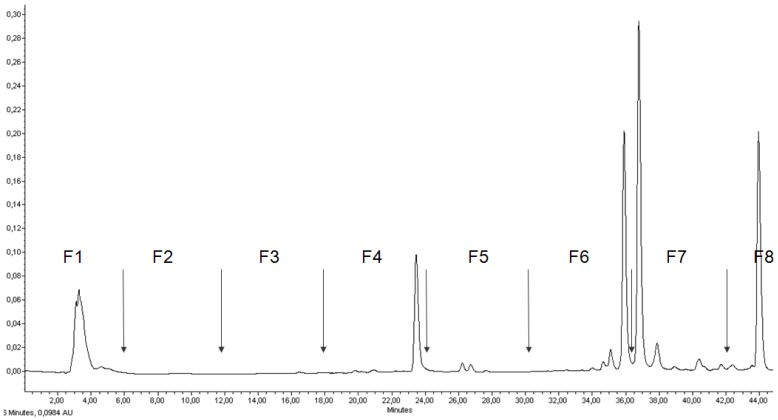
RP-HPLC chromatogram at 435 nm of *Cp* Ethanol extract 2. Definition of the eight fractions to collect by flash liquid chromatography.

Figure 1 presents the Ethanol extract 2 RP-HPLC chromatogram at 435 nm, with the definition of the fractions tested in the three cancer cell lines. Ethanol extract 2 contained four major and 13 minor molecules detected at 435 nm. The F1 peak was a mix of several unseparated polar molecules, including at least one major pigment. The collected fractions were green, gray, yellow or colorless.

Table 2 presents the antiproliferative activity of the eight fractions in the three cancer cell lines (72 h, 100 µg·mL^−1^ in cell culture medium).

**Table 2:** Growth inhibition (%) after a 72 h treatment with 100 µg·mL^−1^ *Cp* fractions obtained from flash chromatography of the Ethanol 2 extract. *t* test (treated *vs*. control): * *p <* 0.05, ** *p <* 0.01. Negative values indicate stimulation of cell growth.

	Cell line		
	A-2058 Malignant melanoma	A-549 Lung adenocarcinoma	MCF-7 Mammary carcinoma
Flash chromatography F1	−2.9 ± 0.3	1.6 ± 0.2	7.9 ± 0.1
Flash chromatography F2	19.2 ± 0.2	5.2 ± 0.2	12.4 ± 0.1
Flash chromatography F3	91.2 ± 0.1 *	54.4 ± 0.2 **	36.8 ± 0.1 *
Flash chromatography F4	34.4 ± 0.2 **	45.7 ± 0.2 **	29.0 ± 0.1 *
Flash chromatography F5	93.0 ± 0.1 *	19.2 ± 0.2 **	**36.1 ± 0.1**
Flash chromatography F6	21.3 ± 0.2 *	17.6 ± 0.2 **	57.3 ± 0.2 *
Flash chromatography F7	5.0 ± 0.2	−4.7 ± 0.2	−2.6 ± 0.2
Flash chromatography F8	0.5 ± 0.3	−26.9 ± 0.2	−10.5 ± 0.2

Fractions 3–6 were identified as the most active, significantly inhibiting the growth of the three cancer cell lines at 100 µg·mL^−1^. Interestingly, F3 and F5 inhibited more than 90% of the A-2058 melanoma cells growth at 100 µg·mL^−1^.

### 2.3. Cytotoxic and Pro-Apoptotic Activity of F3 and F5

**Figure 2:**
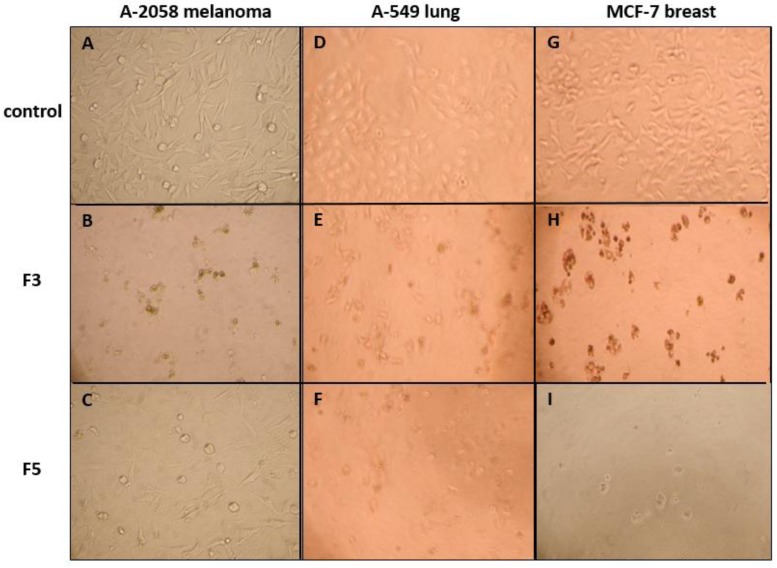
Morphological changes in A-2058 melanoma cells, A-549 lung carcinoma cells and MCF-7 breast adenocarcinoma cells after a 72 h exposition to a control cell culture medium (**A**, **D**, **G**) or to a medium containing 100 µg·mL^−1^ of F3 (**B**, **E**, **H**) or F5 (**C**, **F**, **I**). Condensation and fragmentation into apoptotic bodies unequivocally demonstrated the strong cytotoxicity of F3 and F5 and suggested their pro-apoptotic effect.

**Figure 3:**
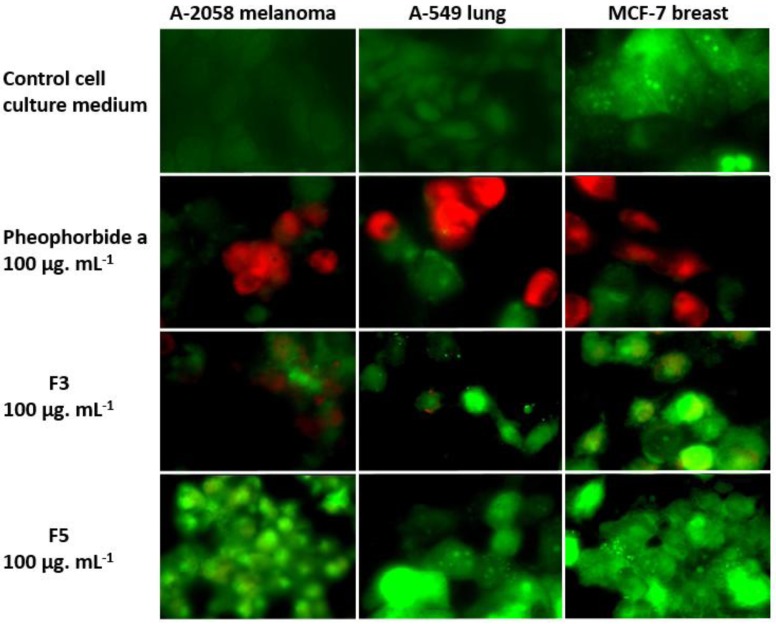
F3 100 µg·mL^−1^ induces apoptosis of A-2058 melanoma cells, A-549 lung adenocarcinoma cells and MCF-7 mammary carcinoma cells. The red labeling by Annexin-V-Alexa 568 indicates phosphatidylserines exposure on the external leaflet of the cytoplasmic membrane, characteristic of early apoptosis.

The cytotoxicity and pro-apoptotic activity of F3 and F5 were confirmed in the three cancer cell lines by the observation of cell condensation and blebbing (Figure 2), and demonstration of phosphatidylserines exposure on the external leaflet of the cancer cells cytoplasmic membrane, characteristic of early apoptosis (Figure 3).

### 2.4. Characterization of the *Cp* Fractions

**Figure 4:**
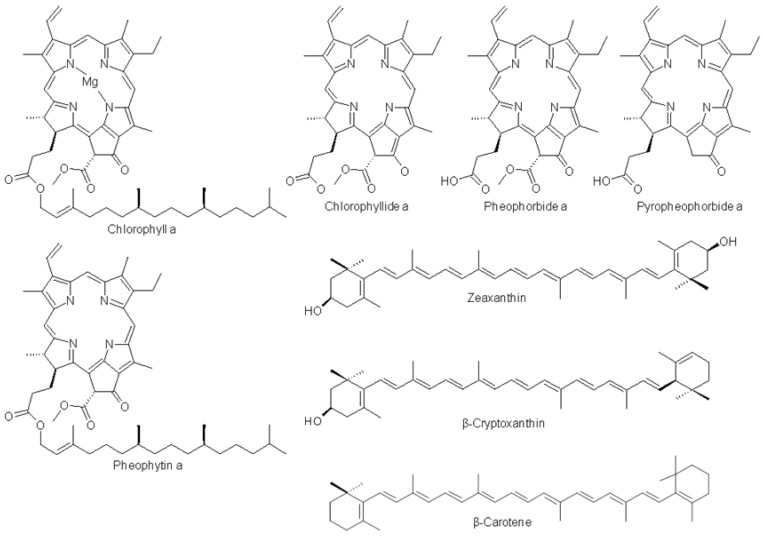
Chemical structures of pigments identified in the *Cp* Ethanol 2 extract.

Pigment characterization in the *Cp* fractions was based on the cross-check analysis of polarity, pigmentation, absorption spectra, maximal absorption wavelengths, band III/II ratios, coherence of the presence of the pigment in *Cp*, high resolution mass spectrometric data, and when possible, comparison with pigments standards, as recommended in the Jeffrey monograph [10] (Table 3, Table 4, Table 5 and Table 6, and supplement data for UV-vis, HRMS and HR MSMS spectra in Supplementary Materials). The chemical structures of pigments identified in the *Cp* Ethanol 2 extract are presented in Figure 4.

**Table 3:** UV-vis characteristics of standard pigments as measured in the HPLC conditions.

Standard Pigment	λ_max_ (nm)			Ratio
	I	II	III	III/II
Chlorophyllide a	-	434.0	668.0	-
Pheophorbide a	-	410.0	665.4	-
Pyropheophorbide a	-	407.6	660.4	-
Zeaxanthin	-	454.8	481.6	32%
β-cryptoxanthin	-	447.3	474.9	23%
Chlorophyll a	-	429.3	660.5	-
Pheophytin a	-	409.0	666.0	-
β-carotene	-	453.6	481.6	25%

**Table 4:** Tentative identification of *Cp* pigments based on chromatographic, UV-vis and polarity characteristics of HPLC peaks contained in the *Cp* Ethanol 2 extract flash chromatography fractions.

Fraction	Peak	HPLC Rt (min)	λ_max_ (nm)			Ratio	Tentative Identification
			I	II	III	III/II	
**F1**	**1**	3.5		430.0	666.7	-	**Chlorophyllide a**
**F2**	**2**	9.3		408.8	666.7	-	**Chlorophyllone a**
**F3**	**3**	12.5		410.0	666.7	-	**Pheophorbide a**
**F4**	**4**	21.0		410.0	660.0	-	**Pyropheophorbide a**
	**5**	24.0		454.9	481.7	28%	**Zeaxanthin**
**F5**	**6**	26.4		447.6	474.4	46%	**5,6-epoxy β-cryptoxanthin**
	**7**	26.9		447.9	474.4	20%	**β-cryptoxanthin**
**F6**	**8**	34.7		429.4	666.7	-	**Chlorophyll a allomer**
	**9**	35.0	433.0	451.2	485.3	50%	**Unidentified carotenoid**
	**10**	36.0		429.4	661.7	-	**Chlorophyll a**
**F7**	**11**	36.8		430.6	663.0	-	**Chlorophyll a epimer**
	**12**	37.7		417.3	661.7	-	**DV-pheophytin a**
	**13**	39.0		429.4	454.9	40%	**Mutatochrome**
	**14**	40.5		408.8	669.1	-	**MV-pheophytin a**
	**15**	41.4		401.5	669.1	-	**Purpurin-7 phytyl ester**
	**16**	42.3		410.0	666.7	-	**Pheophytin a**
**F8**	**17**	43.6		454.9	480.4	23%	**β-carotene**

#### 2.4.1. F1

The major peak in F1 exhibited a non-Gaussian distribution, suggesting that it could contain several polar molecules poorly retained and not separated by the C18 column. The green color of F1, absorption maxima (430 and 660 nm) and polarity suggested that F1 contained chlorophyllide a [10]. This identification was unequivocally confirmed by the HRMS comparative analysis of F1 with standard chlorophyllide a (Table 5 and Table 6).

#### 2.4.2. F2

The absorbance maxima of F2 at 409 and 665 nm, and high polarity, suggested it contained chlorophyllone a [10,11], for which no standard is commercially available for HRMS analysis. HRMS analysis allowed the detection of ions corresponding to those obtained with the chlorophyllide a standard. However, as F2 did not present any absorbance maximum at 430 nm, the presence of chlorophyllide a was excluded. Further HRMS analysis with purified chlorophyllone a will be necessary to confirm that the detected masses correspond to chlorophyllone a relative ions.

**Table 5:** HRMS of commercially available standard pigments suspected in *Cp* flash chromatography fractions.

Standardpigment	Formula	UPLC Rt (min)	Theorical*m/z*				Experimental*m/z*						
			M^.^	[M + H]^+^	[M + Na]^+^	[M − Mg + 3H]^+^	M^.^	[M + H]^+^	[M + Na]^+^	[M − Mg + 3H]^+^	Fragments		
**Chlorophyllide a**	C_35_H_34_N_4_O_5_Mg	7.08	-	615.2458	-	593.2764	-	615.2580	-	593.2761	533.2545	-	-
**Pheophorbide a**	C_35_H_36_N_4_O_5_	7.05	-	593.2764	-	-	-	593.2762	-	-	533.2544	539.2417	-
**Pyropheophorbide a**	C_33_H_34_N_4_O_3_	7.45	-	535.2709	557.2529	-	-	535.2699	557.2520	-	435.2537	256.3005	-
**Zeaxanthin**	C_40_H_56_O_2_	8.14	568.4280	-	-	-	568.4268	-	-	-	549.4098	-	-
**β-Cryptoxanthin**	C_40_H_56_O	10.04	-	553.4409	575.4137	-	-	553.4346	-	-	533.4146	460.3685	-
**Chlorophyll a**	C_55_H_72_N_4_O_5_Mg	10.03	-	893.5432	915.5251	871.5737		893.5403	915.5237	871.5727	614.2376	555.2232	637.2258
**β-Carotene**	C_40_H_56_	10.83	536.4382	-	-	-	536.4370	-	-	-	444.3751	-	-

**Table 6:** HRMS of pigments contained in the *Cp* Ethanol 2 extract flash chromatography fractions and confirmation of *Cp* pigment composition.

Fractions	UPLC Rt (min)	Experimental *m/z*							Confirmation of pigment identification
	-	M^.^	[M + H]^+^	[M + Na]^+^	[M − Mg + 3H]^+^	Fragments			
F1	7.08	-	-	-	593.2743	533.2540	-	-	Chlorophyllide a
F2	7.08	-	-	-	593.2743	533.2540	-	-	Lack of chlorophyllone a standard to confirm that the detected ions derive from it
F3	7.05	-	593.2756	-	-	533.2542	-	-	Pheophorbide a
F4	7.45	-	535.2702	557.2519	-	435.2534	-	-	Pyropheophorbide a Zeaxanthin
	8.15	568.4260	-	-	-	549.4086	-	-	
F5	10.05	-	-	575.4137	-	371.2270	-	-	β-cryptoxanthin Lack of 5,6-epoxy-β-cryptoxanthin standard to confirm that the detected ions derive from it
F6	10.04	-	893.5405	915.5229	871.5722	614.2380	555.2234	637.2262	Chlorophyll a Pheophytin a (pheophytination of chlorophyll a) No confirmation of the presence of the unidentified carotenoid
	10.61		871.5724	-	-	533.2548	-	-	
F7	10.04	-	893.5405	915.5229	871.5722	614.2380	555.2234	637.2262	Chlorophyll a epimer Pheophytin a No confirmation of the presence of DV- and MV-pheophytin a, mutatochrome and purpuryl-7-phytyl ester
	10.62	-	871.5723	893.5538	-	533.2539	-	-	
F8	10.80	536.4372	-	-	-	-	-	-	β-carotene

#### 2.4.3. F3

F3 contained polar molecules eluating in the first third of the flash chromatography gradient (Figure 1), and was gray/green, suggesting the presence of pheophorbide derivatives [10]. It did not contain major *Cp* pigments but was very cytotoxic in the three cancer cell lines, at 100 µg·mL^−1^ (Figure 2). The absorbance maxima at 410 and 666 nm confirmed that F3 contained a phorbin-type molecule. The major ion contained in the HRMS spectrum in positive mode was observed at *m/z* 593.2756, and unequivocally identified as the [M + H]^+^ ion of pheophorbide a, in coherence with the polarity and cytotoxicity of F3 [12,13]. The fragment ion at 533.2542 confirmed the identification of pheophorbide a. The presence of minor pheophorbide a derivatives in F3 is not excluded as the pro-apoptotic activity of F3 at 100 µg·mL^−1^ was less pronounced than that of standard pheophorbide a100 µg·mL^−1^, used as a positive apoptosis inducer in our experiment (Figure 3). As pheophorbide a is a chlorophyll a degeneration product, that should not be present in a living cell, its presence in the *Cp* pigment composition probably reflects the chlorophyll a degradation during the pigment extraction process rather than its real presence in *Cp* living cells.

#### 2.4.4. F4

F4 contained one major peak corresponding to a major *Cp* pigment, and one minor pigment, as revealed by the RP-HPLC analysis. The absorbance maxima of F4 at 410 and 660 nm, together with its olive green color, suggested that at least one pigment was a chlorophyll derivative. The absorption spectrum performed on the major peak unambiguously revealed it was a carotenoid, with absorbance maxima at 455 and 482 nm, suggesting it could be alloxanthin, β,β-carotene, diatoxanthin or zeaxanthin. β,β-carotene was excluded as it is very apolar and cannot be present in F4 according to the flash chromatography gradient. Alloxanthin was excluded as this carotenoid is characteristic of cryptophytes [10]. The band III:II ratio was calculated as 29%, a value close to that described for zeaxanthin (23%) and diatoxanthin (26%) in HPLC eluants [10]. Zeaxanthin and diatoxanthin have very close polarities, coherent with the polarity of the major F4 carotenoid. Diatoxanthin is present in diatoms, prymnesiophytes, chrysophytes and dinoflagellates but has never been described in glaucophytes. In contrast, zeaxanthin is described as one of the major *Cp* carotenoid [10]. Identification of the F4 carotenoid as zeaxanthin was confirmed by the HRMS study in positive mode which gave a major ion at *m/z* 568.4260 and a fragment at *m/z* 549.4086. These values were coherent with those obtained with standard zeaxanthin and the *m/z* 568.4260 ion was identified as the radical cation of zeaxanthin [14]. We also checked that the F4 carotenoid was not lutein (*m/z* 568, close polarity). This hypothesis was confirmed because lutein presents different absorption maxima (448 and 477 nm in HPLC eluant), different band III/II% ratio (54% in HPLC eluant), and is only described in red seaweeds, green algae and higher plants [10]. In order to know if the antiproliferative activity of F4 was due to the presence of zeaxanthin, the antiproliferative activity of standard zeaxanthin (Sigma-Aldrich, Saint-Quentin Fallavier, France) was studied in A-2058 melanoma cells. This study revealed that 100 µg·mL^−1^ zeaxanthin induced 38.4% ± 4.2% growth inhibition at 72 h (*n =* 4, *p =* 0.0245, statistically significant in the *t test*), demonstrating for the first time the antimelanoma activity of zeaxanthin, complementary to its already known ability to inhibit PDGF-induced migration of dermal fibroblasts in the skin [15]. This value was coherent with the antiproliferative activity of the F4 fraction (34.4% ± 0.2% growth inhibition at 72 h at 100 µg·mL^−1^). It is however important to notice that the standard zeaxanthin weakly solubilized in the aqueous cell culture medium, as revealed by microscopic observations of zeaxanthin precipitates after dilution in the cell culture medium of a 10^−3^ M stock solution prepared in DMSO, ethanol or methanol. Further studies are currently underway in our lab to make precise its IC_50_ in melanoma cells. The minor chlorophyll derivative contained in F4 absorbed at 410 and 660 nm, was slightly more apolar than pheophorbide a, and was unequivocally identified as pyropheophorbide a by HRMS (Table 5 and Table 6). Pyropheophorbide a, in spite of its minor abundance in F4, may participate to the antiproliferative activity of F4 in melanoma cells, as some derivatives of this molecule are used for tumor dynamic phototherapy [16].

#### 2.4.5. F5

F5 contained two peaks corresponding to molecules with a median polarity as they eluated in the middle of the flash chromatography gradient. The yellow color and absorption spectra of the two molecules indicated they were carotenoids with very close absorption maxima and polarities, but different band III/II ratios (λ_max_ molecule 1 = 447.6 and 474.4 nm; III/II: 46%) and (λ_max_ molecule 2 = 448.0 and 474.0 nm; III/II: 20%). According to their absorption maxima, band III/II ratio, polarity and presence in *Cp* [17,18], the two carotenoids were tentatively identified as 5,6-epoxy-β-cryptoxanthin [19] and β-cryptoxanthin. HRMS analysis of F5 and standard β-cryptoxanthin confirmed the identification of β-cryptoxanthin with a major ion observed at *m/z* 575.4137 ([M + Na]^+^) and a fragment at *m/z* 371.2270. The identification of 5,6-epoxy-β-cryptoxanthin could not be unequivocally confirmed because of the lack of standard.

#### 2.4.6. F6

F6 contained one major peak and two minor peaks. The major peak corresponded to a major *Cp* pigment and was identified as chlorophyll a on the basis of its absorption spectrum (λ_max_ = 429.4 and 661.7 nm), polarity, and abundance [10]. The HRMS profile of F6, compared with that of standard chlorophyll a, confirmed this identification (Table 5 and Table 6). The HRMS detection of ions corresponding to pheophytin a may be explained by a partial pheophytination of chlorophyll a in the F6 sample and/or in the mass spectrometer source. The first minor peak corresponded to a polar derivative of chlorophyll a with absorption maxima at 429.4 and 661.7 nm and was identified as chlorophyll a allomer [10,20,21], in agreement with the HRMS analysis. The second minor peak corresponded to an unidentified carotenoid, according to its absorption maxima (λ_max_ = 433.0, 451.2 and 485.3 nm; III/II: 50%). The presence of this carotenoid was not confirmed by HRMS analysis, which only gave ions corresponding to chlorophyll a and pheophytin a.

#### 2.4.7. F7

F7 was green, contained one major *Cp* pigment and five minor peaks, but had low cytotoxic activity in cancer cells. The major F7 *Cp* pigment presented absorption maxima at 430.6 and 663.0 nm, was slightly more apolar than chlorophyll a, and was identified as chlorophyll a epimer. HRMS analysis confirmed the presence of chlorophyll a related ions in F7 (Table 6). The first minor peak on the F7 chromatogram exhibited absorption maxima at 417.3 and 661.7 nm. The only chlorophyll derivative presenting a λ_max_ at 417 nm and slightly more apolar than chlorophyll a is divinyl pheophytin a [10,22]. This identification could not be confirmed by HRMS because no DV-pheophytin a standard was available. The second peak corresponded to a minor carotenoid with λ_max_ = 406.0, 429.4 and 454.9 nm; III/II: 40%, identified as mutatochrome (5,8-epoxy-β,β-carotene), according to its polarity, absorption spectrum, and band III/II ratio [19]. Although no mutatochrome standard was available to confirm this identification by HRMS, the presence of mutatochrome in *Cp* is coherent because it is a β,β-carotene derivative. The third minor peak absorbed at λ_max_ = 408.9 and 669.1 nm, and was identified as monovinyl pheophytin a according to its absorption maxima and polarity [10,22]. No MV-Pheophytin a standard was available for HRMS confirmation. The fourth minor peak was most likely identifiable as purpurin-7-phytyl ester according to its polarity and absorption maxima (λ_max_ = 401.5 and 669.1 nm) [23]. No standard was available for HRMS confirmation and HRMS study of F7 did not allow confirmation of the presence of purpurin-7-phytyl ester relative ions. It is most probable that the presence of purpurin-7-phytyl ester in F7 indicates the oxidative transformation of a porphyrin during the pigment extraction process, rather than its real presence in living cells. The fifth minor peak was unequivocally identified as pheophytin a, according to its polarity, absorption maxima, and HRMS spectrum (Table 5 and Table 6 and [10]).

#### 2.4.8. Fraction 8

F8 only contained a major *Cp* pigment, unequivocally identified as β,β-carotene, according to its color, absorption maxima, hydrophobicity, band III/II ratio, abundance in *Cp* [10], and HRMS analysis (Table 5 and Table 6).

## 3. Experimental Section

### 3.1. Chemicals, Reagents and Chromatography Columns

Pigments standards were obtained from DHI Lab Denmark and Sigma-Aldrich France. Ultra-pure water was obtained using a Milli-Q system (Millipore, Molsheim, France). All reagents were of HPLC grade. Flash chromatography columns were obtained from Interchim, Montluçon, France and RP-HPLC columns from Phenomenex, Le Pecq, France.

### 3.2. Microalgae

*Cyanophora paradoxa* (*Cp*) is a freshwater glaucophyte containing two chloroplasts (cyanelles), relics of cyanobacterial endosymbionts, as demonstrated by their thylakoid organization and the presence of peptidoglycan layers separating the cyanelle from the cytoplasmic content of the protist host cell. It has been widely studied as a model species to understand how oxygenic photosynthesis was established in eukaryotes and how the transfer of endosymbiont genes to the protist host cell allowed plastid establishment and conservation. The pigments described in *Cp* are chlorophyll *a*, β-carotene, allo-phycocyanin, c-phycocyanin, β-cryptoxanthin, and zeaxanthin [18,24,25].

### 3.3. Microalgae Culture, Collection and Storage

**Figure 5:**
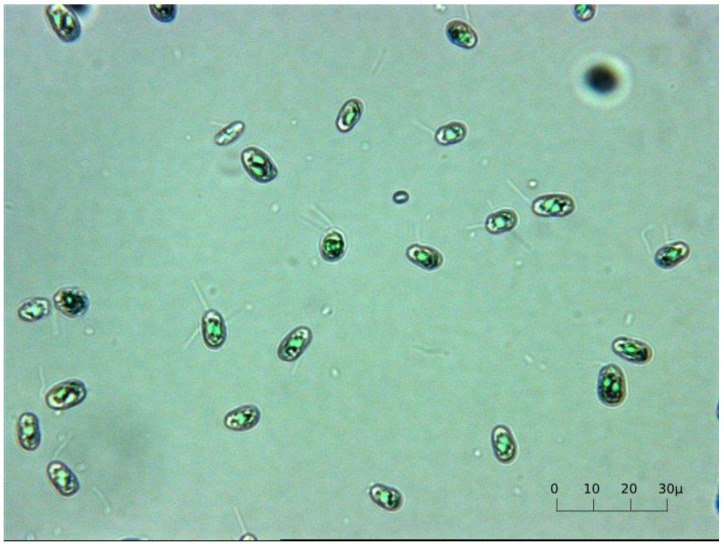
The starting *Cyanophora paradoxa* culture at IFREMER PBA Nantes.

The xenic strain *Cyanophora paradoxa* SAG 29.80 (SAG culture collection, University of Göttingen, Germany) was cultivated at IFREMER PBA, Nantes, in 10L flasks under continuous illumination at an average light intensity of 180 µmol·m^−2^·s^−1^ (Figure 5). Growth was performed at 20 °C, in pH unregulated batch culture, in Walne (Conway) medium diluted in 0.22 µm sterile-filtered natural freshwater. The cell suspension was harvested at the end of the exponential growth phase, and cells were separated from culture medium by soft centrifugation (4000× *g*, 20 min, 10 °C). Cells were frozen at −20 °C, sent to laboratory LIENSs, La Rochelle, and freeze-dried at −55 °C and P *<* 1 hPa, on a freeze-dryer equipped with a HetoLyoPro 3000 condenser and Heto cooling trap (Thermo Scientific, Villebon sur Yvette, France).

### 3.4. Pigments Extraction, Fractionation and Purification

**Figure 6:**
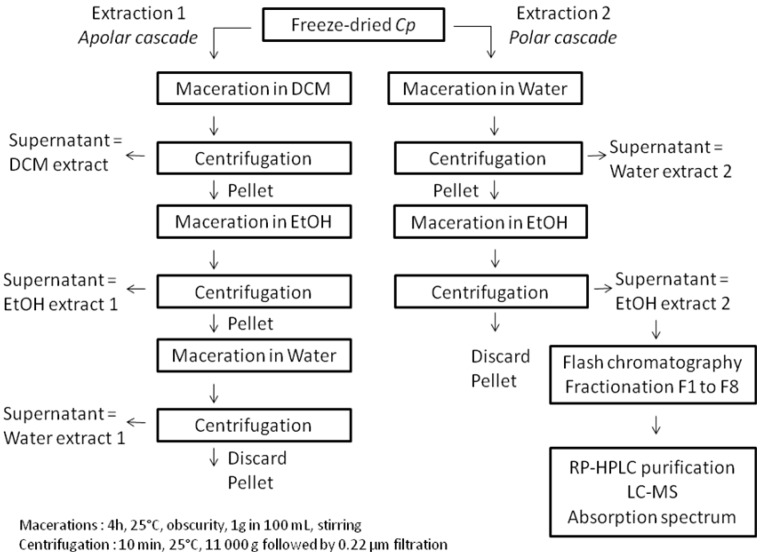
The pigments extraction and purification sequence. DCM: dichloromethane, EtOH: Ethanol, Water: Ultrapure water.

The pigments extraction sequence is described in Figure 6. Two extractions referred to as apolar and polar cascades were performed from the freeze-dried microalgae powder, to obtain a dichloromethane extract, two ethanol extracts and two water extracts. The polar cascade was performed to recover phycocyanin in the water extract 2 and remove it from the following ethanol extract 2 and flash chromatography fractions. The biological activity of *Cp* phycocyanin is currently under study in our lab and will be discussed later. Flash liquid chromatography fractionation was performed from the ethanol extract 2, using an Interchim Puriflash PF430 system. One mL of *Cp* ethanol extract 2 (0.25 g·L^−1^) was added to the top of a PF-C18 column (20 g, 5 µm). The column was then washed with a mobile phase consisting of a ternary gradient of solvent A (Methanol/water (80/20)); solvent B (Acetonitrile/water (90/10)) and solvent C (ethyl acetate). The gradient flow program was set as follows: 0 min—100% A, 3 min—100% B, 35 min—30% B and 70% C, 38 min—100% C, 41 min—100% C, 43 min—100% B, 45 min—100% A. The flow rate was 5 mL·min^−1^ and elution was monitored from 400 to 800 nm, with an automatic collector (20 mL per tube).

### 3.5. RP-HPLC Pigments Analysis

The number of molecules contained in flash chromatography fractions and their absorption spectra were determined using an analytical RP-HPLC system composed of a binary pump (Waters, W600), an autosampler (Waters, W717), a thermostated (20 °C) column compartment, and a photodiode-array detector (Waters, W486). A 100 µL sample was injected into a Phenomenex Luna C18 (2) analytical column (250 × 4.6 mm, 10 µm). The mobile phase and gradient flow program were simplified compared to flash chromatography to obtain a quick separation. The mobile phase consisted of a binary gradient of solvent A (methanol/water (80/20)) and solvent B (ethyl acetate) and the gradient flow was set as follows: 0 min—100% A, 8 min—95% A, 5% B, 9 min—100% B, 13 min—100% A, and 15 min—100% A. The flow rate was 1 mL·min^−1^ and elution was monitored at 435 nm. Purification of single peaks for mass spectrometry was also performed in semi-preparative conditions on a Phenomenex Luna C18 (2) column (250 × 10.0 mm, 10 µm) with a flow rate fixed at 5 mL·min^−1^ and with the same gradient and detection conditions.

### 3.6. Mass Spectrometry

Mass spectrometry analyses were performed using an Acquity UPLC (Waters, Milford, MA, USA) coupled to a xevo G2 S Q-TOF mass spectrometer (Waters, Manchester, UK). Ten microliters of sample were injected into a Waters Acquity UPLC BEH C18 column (2.1 × 50 mm, 1.7 µm), at 25 °C. Separation consisted of a gradient of solution A (0.01% formic acid (FA), 0.05% ammonia in water) in solution B (0.01% FA in acetonitrile) and C (0.01% FA in ethyl acetate) at a flow rate of 200 µL·min^−1^ as follows: 0–0.2 min, 2% B; 0.2–5.2 min, 2%–100% B; 5.2–5.70 min, 100% B; 5.70–10.70 min, 100% B–100% C; 10.70–11.20 min, 100% C; 11.20–11.25 min, 100% C–100% B; 11.25–11.50 min, 100% B; 11.50–11.55 min, 100%–2% B; 11.55–15 min, 2% B. ESI conditions were: source temperature 120 °C, desolvation temperature 500 °C, cone gas flow 50 L·h^−1^, desolvation gas flow 1000 L·h^−1^, capillary voltage 0.5 kV, sampling cone 35 and Source offset 80. The instrument was set to acquire over the *m/z* range 50–950 with scan time 0.15 s. Data were collected in the positive (ESI+) electrospray ionization mode, in MS^E^ continuum mode. Leu-enkephalin (MW = 555.62 Da) (1 ng·µL^−1^) was used as lock mass. The mass spectrometer was calibrated before analyses using a 0.5 mM sodium formate solution.

### 3.7. Cell Culture and Viability Assay

MCF-7 human mammary carcinoma cells, A549 human lung adenocarcinoma cells, and A2058 human melanoma cells (LGC ATCC Standards, Molsheim, France) were grown as monolayers, at 37 °C in a 5% CO_2_–95% air humidified atmosphere, in DMEM (Fischer scientific, Illkirch, France) supplemented with 10% heat-inactivated (56 °C, 30 min) FCS (Dutscher, Brumath, France) to which were added penicillin 100 U·mL^−1^ and streptomycin 100 µg·mL^−1^. The algal extracts and related fractions were evaporated to dryness using a Büchi rotavapor and solubilized in DMSO before being diluted in the cell culture medium to a final concentration of 100 µg·mL^−1^. The final DMSO concentration was lower than 1% and tested as a negative control. Cell viability at 72 h was studied using the MTT assay in 96-well microplates as previously described [26,27].

### 3.8. Apoptosis Assay

Five thousand cells were grown for 24 h on epifluorescence live cell array slides (Nunc, Dutscher, France) and treated for 72 h with control culture medium (negative control), culture medium containing 100 µg·mL^−1^ pheophorbide a (positive apoptosis control), or microalgae fractions diluted to 100 µg·mL^−1^ in cell culture medium. Phosphatidylserines translocation onto the outer side of the plasma membrane of early apoptotic cells was detected using an Annexin V-Alexa-568 fluorochrome (Life Technologies SAS, Saint-Aubin, France), and at the same time DNA was labeled using the cell-permeant Syto^®^ green fluorescent nucleic acid stain kit (Life Technologies SAS, Saint-Aubin, France). Cells were incubated for 4 h min at 37 °C with the labeling mix solution, and observed using a Leica epifluorescence microscope, equipped with an I3 epifluorescence filter block (blue excitation 450–490 nm) and a numeric camera. Control cells were labeled in green with a round shaped nucleus while apoptotic cells, having exposed phosphatidylserines, appeared as red spots. Some early apoptotic cells also exhibited a mixed green and red fluorescence, the red fluorescence being located in the cytoplasmic membrane.

### 3.9. Statistical Analysis

The significativity of *Cp* extracts cytotoxicy was determined using the unpaired Student *t* test (*n =* 3, triplicate independent cytotoxicity assays).

## 4. Conclusions

This study establishes the high antiproliferative activity of pigments from *Cyanophora paradoxa* in human melanoma, breast and lung cancer cells, and presents an updated analysis of *Cp* pigment composition, suggesting the presence of mutatochrome, 5,6-epoxy-β-cryptoxanthin and an unidentified carotenoid in this species. The major result of this study is the demonstration and first report that zeaxanthin and β-cryptoxanthin exert a strong antiproliferative activity in A-2058 melanoma cells. As zeaxanthin and β-cryptoxanthin antiproliferative activity has already been documented in other cancer cells [28,29,30], our data obtained in melanoma cells confirm that these pigments may be of interest to prevent or treat cancer.

## Supplementary Files

Supplementary File 1 Supplementary Materials (PDF, 1462 KB)

## References

[1] Talero E., Avila-Roman J., Motilva V. (2012). Chemoprevention with phytonutrients and microalgae products in chronic inflammation and colon cancer. Curr. Pharm. Des..

[2] Nappo M., Berkov S., Massucco C., di Maria V., Bastida J., Codina C., Avila C., Messina P., Zupo V., Zupo S. (2012). Apoptotic activity of the marine diatom *Cocconeis scutellum* and eicosapentaenoic acid in BT20 cells. Pharm. Biol..

[3] Guedes A.C., Amaro H.M., Malcata F.X. (2011). Microalgae as sources of carotenoids. Mar. Drugs.

[4] Bhatnagar I., Kim S.-K. (2010). Immense essence of excellence: marine microbial bioactive compounds. Mar. Drugs.

[5] Leya T., Rahn A., Lütz C., Remias D. (2009). Response of arctic snow and permafrost algae to high light and nitrogen stress by changes in pigment composition and applied aspects for biotechnology. FEMS Microbiol. Ecol..

[6] Gagez A.-L., Thiéry V., Pasquet V., Cadoret J.-P., Picot L. (2012). Epoxycarotenoids and cancer. Review. Curr. Bioact. Compd..

[7] Pasquet V., Morisset P., Ihammouine S., Chepied A., Aumailley L., Berard J.-B., Serive B., Kaas R., Lanneluc I., Thiery V. (2011). Antiproliferative activity of violaxanthin isolated from bioguided fractionation of dunaliella tertiolecta extracts. Mar. Drugs.

[8] Moreau D., Tomasoni C., Jacquot C., Kaas R., Le Guedes R., Cadoret J.-P., Muller-Feuga A., Kontiza I., Vagias C., Roussis V. (2006). Cultivated microalgae and the carotenoid fucoxanthin from Odontella aurita as potent anti-proliferative agents in bronchopulmonary and epithelial cell lines. Environ. Toxicol. Pharmacol..

[9] Mathieu V., de Lassalle E.M., Toelen J., Mohr T., Bellahcène A., van Goietsenoven G., Verschuere T., Bouzin C., Debyser Z., de Vleeschouwer S. (2012). Galectin-1 in melanoma biology and related neo-angiogenesis processes. J. Investig. Dermatol..

[10] Jeffrey S., Mantoura R., Wright S., Jeffrey S., Mantoura R., Wright S. (1997). Iternational council of scientific unions. SCOR UNESCO Phytoplankton Pigments in Oceanography: Guidelines to Modern Methods.

[11] Pickering M. (2009). Low Temperature Sequestration of Photosynthetic Pigments: Model Studies and Natural Aquatic Environments. Ph.D. Thesis.

[12] Chan J.Y.-W., Tang P.M.-K., Hon P.-M., Au S.W.-N., Tsui S.K.-W., Waye M.M.-Y., Kong S.-K., Mak T.C.-W., Fung K.-P. (2006). Pheophorbide a, a major antitumor component purified from Scutellaria barbata, induces apoptosis in human hepatocellular carcinoma cells. Planta Med..

[13] Hibasami H., Kyohkon M., Ohwaki S., Katsuzaki H., Imai K., Nakagawa M., Ishi Y., Komiya T. (2000). Pheophorbide a, a moiety of chlorophyll a, induces apoptosis in human lymphoid leukemia molt 4B cells. Int. J. Mol. Med..

[14] Magyar A., Bowman M., Molnar P., Kispert L. (2013). Neutral carotenoid radicals in photoprotection of wild-type Arabidopsis thaliana. J. Phys. Chem. B.

[15] Wu N.-L., Chiang Y.-C., Huang C.-C., Fang J.-Y., Chen D.-F., Hung C.-F. (2010). Zeaxanthin inhibits PDGF-BB-induced migration in human dermal fibroblasts. Exp. Dermatol..

[16] Bellnier D.A., Greco W.R., Loewen G.M., Nava H., Oseroff A.R., Pandey R.K., Tsuchida T., Dougherty T.J. (2003). Population pharmacokinetics of the photodynamic therapy agent 2-[1-hexyloxyethyl]-2-devinyl pyropheophorbide-a in cancer patients. Cancer Res..

[17] Rissler H.M., Durnford D.G. (2005). Isolation of a novel carotenoid-rich protein in *Cyanophora paradoxa* that is immunologically related to the light-harvesting complexes of photosynthetic eukaryotes. Plant Cell Physiol..

[18] Schmidt V., Kies L., Weber A. (1979). Die Pigmente von *Cyanophora paradoxa*, *Gloechaete wittrockiana* und *Glaucocystis nostochinearum*. Arch. Protistenk.

[19] De Rosso V.V., Mercadante A.Z. (2005). Carotenoid composition of two Brazilian genotypes of acerola (*Malpighia punicifolia* L.) from two harvests. Food Res. Int..

[20] Yamauchi N., Harada K., Watada A. (1997). *In vitro* chlorophyll degradation in stored broccoli (*Brassica oleracea* L. 6ar. italica Plen.) florets. Postharvest Biol. Technol..

[21] Zapata M., Rodriguez F., Garrido J. (2000). Separation of chlorophylls and carotenoids from marine phytoplankton: A new HPLC method using a reversed phase C8 column and pyridine-containing mobile phases. Mar. Ecol. Progr. Ser..

[22] Smith A., Witty M. (2002). Heme, Chlorophyll, and Bilins: Methods and Protocols.

[23] Hegazi M., Perez-Ruzafa A., Almela L., Candela M. (1998). Separation and identification of chlorophylls and carotenoids from *Caulerpa prolifera*, *Jania rubens* and *Padina pavonica* by reversed-phase high-performance liquid chromatography. J. Chromatogr. A.

[24] Chapman D.J. (1966). The pigments of the symbiotic algae (cyanomes) of *Cyanophora paradoxa* and *Glaucocystis nostochinearum* and two Rhodophyceae, *Porphyridium aerugineum* and *Asterocytis ramosa*. Arch. Mikrobiol..

[25] Kies L., Kremer B., Margulis L., Corliss J., Melkonian M., Chapman D. (1990). Phylum Gaucocystophyta. Handbook of Protocista.

[26] Mosmann T. (1983). Rapid colorimetric assay for cellular growth and survival: Application to proliferation and cytotoxicity assays. J. Immunol. Methods.

[27] Testard A., Picot L., Lozach O., Blairvacq M., Meijer L., Murillo L., Piot J.-M., Thiéry V., Besson T. (2005). Synthesis and evaluation of the antiproliferative activity of novel thiazoloquinazolinone kinases inhibitors. J. Enzyme Inhib. Med. Chem..

[28] Firdous A.P., Sindhu E.R., Ramnath V., Kuttan R. (2010). Anti-mutagenic and anti-carcinogenic potential of the carotenoid meso-zeaxanthin. Asian Pac. J. Cancer Prev. APJCP.

[29] Cha K.H., Koo S.Y., Lee D.-U. (2008). Antiproliferative effects of carotenoids extracted from Chlorella ellipsoidea and Chlorella vulgaris on human colon cancer cells. J. Agric. Food Chem..

[30] Ferreres F., Pereira D.M., Gil-Izquierdo A., Valentão P., Botelho J., Mouga T., Andrade P.B. (2010). HPLC-PAD-atmospheric pressure chemical ionization-MS metabolite profiling of cytotoxic carotenoids from the echinoderm Marthasterias glacialis (spiny sea-star). J. Sep. Sci..

