# Supplementary material for: Antiproliferative Activity of Cyanophora paradoxa Pigments in Melanoma, Breast and Lung Cancer Cells
Source: Mar Drugs. 2013 Nov 1;11(11):4390–406. doi: 10.3390/md11114390 (PMC3853734; doi:10.3390/md11114390)
Supplement: Supplementary File 1 — Supplementary Materials (PDF, 1462 KB) [file marinedrugs-11-04390-s001.pdf]

Supplementary Materials

Figure S1. High resolution MS (**A**) and MSMS (**B**) spectra of standard chlorophyllide a.

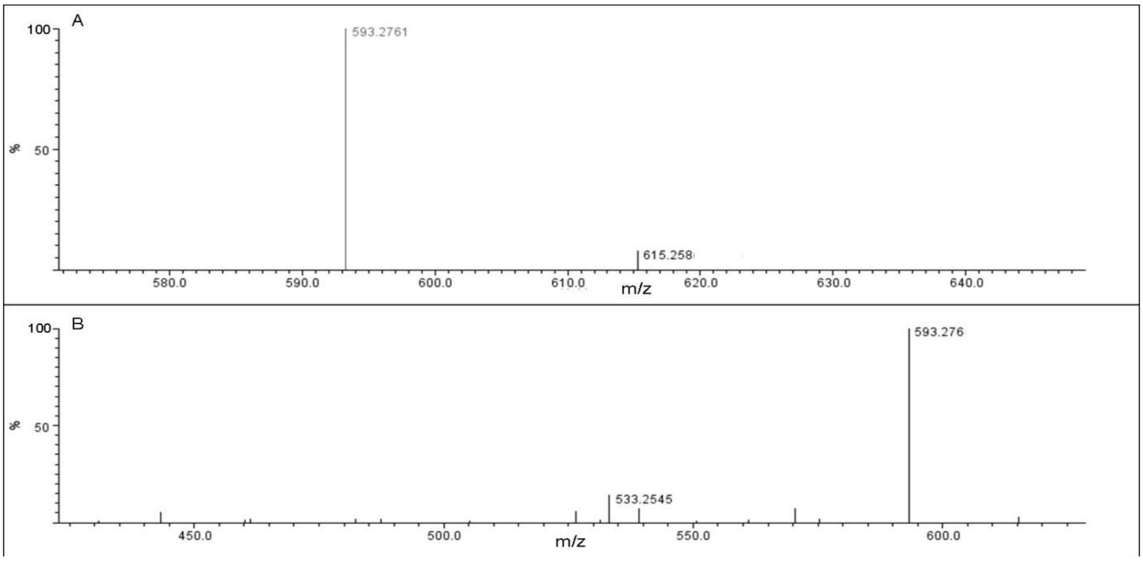

Figure S2. UV-vis (**A**), high resolution MS (**B**) and MSMS (**C**) spectra of Fraction 1 (chlorophyllide a).

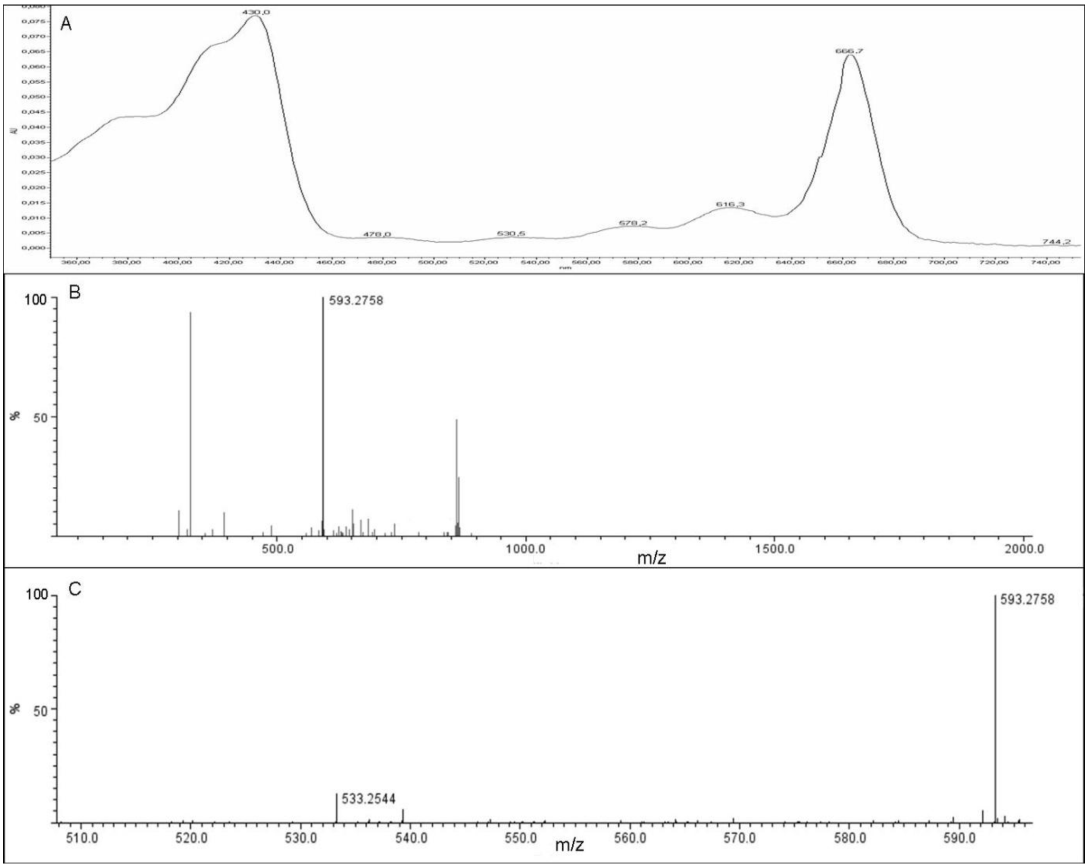

Figure S3. UV-vis (**A**), high resolution MS (**B**) and MSMS (**C**) spectra of Fraction 2 (chlorophyllone).

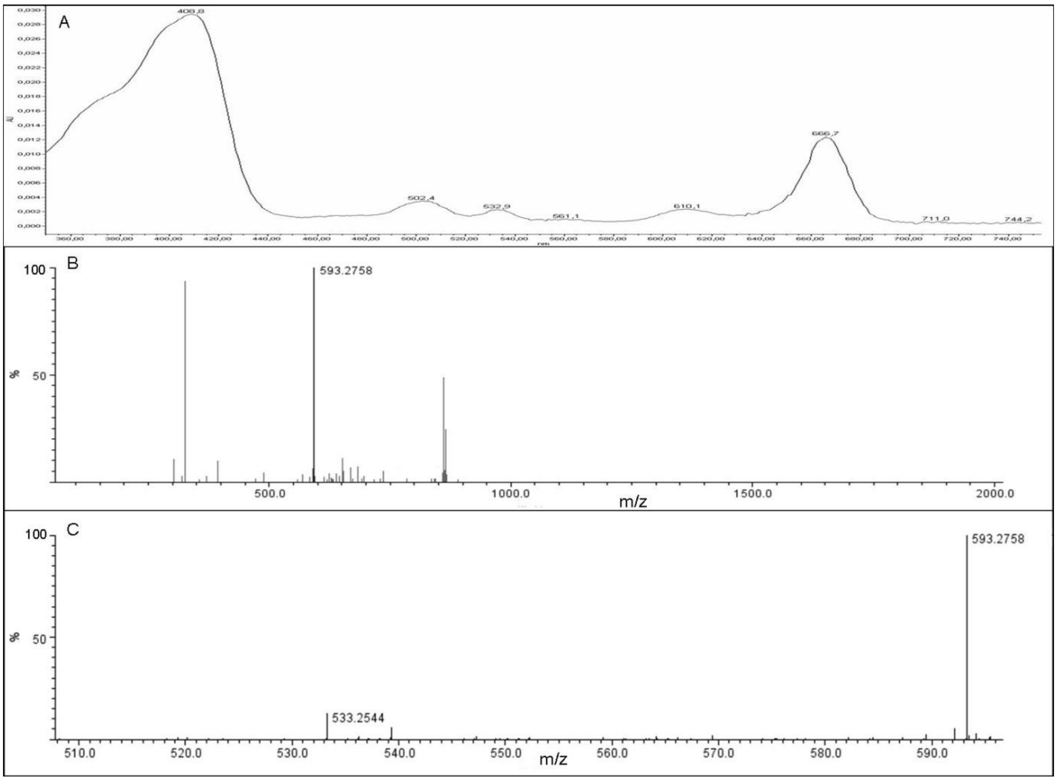

Figure S4. UV-vis (**A**), high resolution MS (**B**) and MSMS (**C**) spectra of standard pheophorbide a.

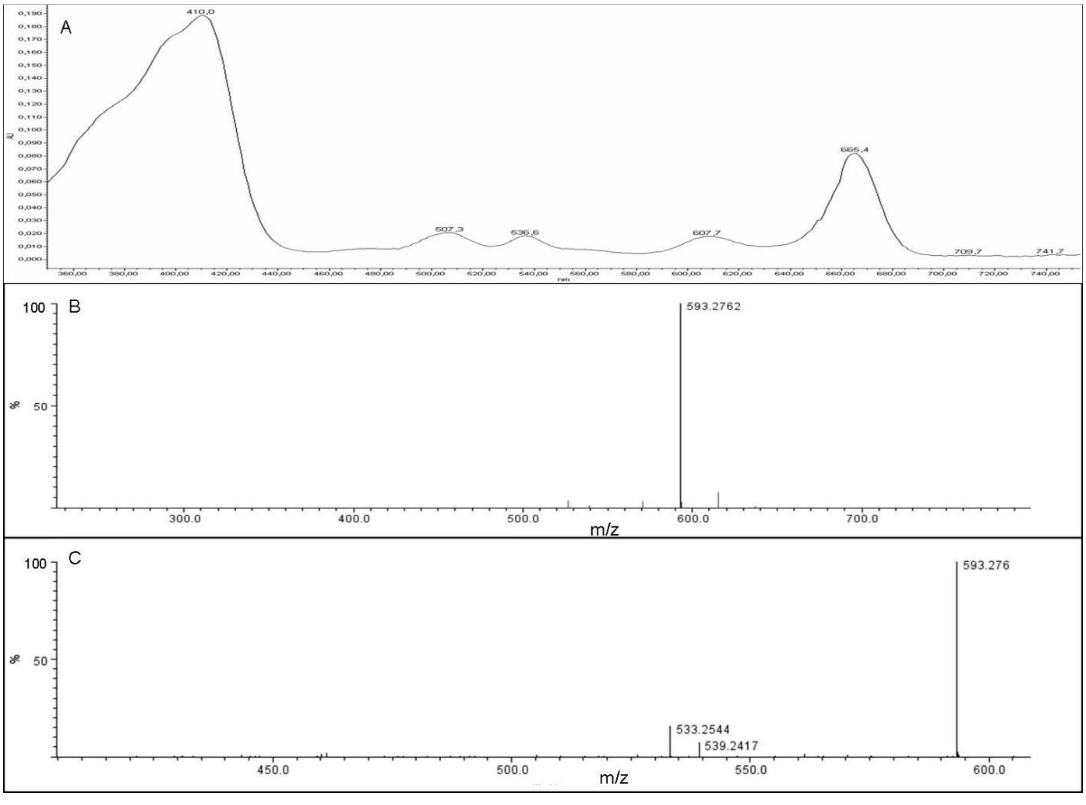

Figure S5. UV-vis (**A**), high resolution MS (**B**) and MSMS (**C**) spectra of Fraction 3 (pheophorbide a).

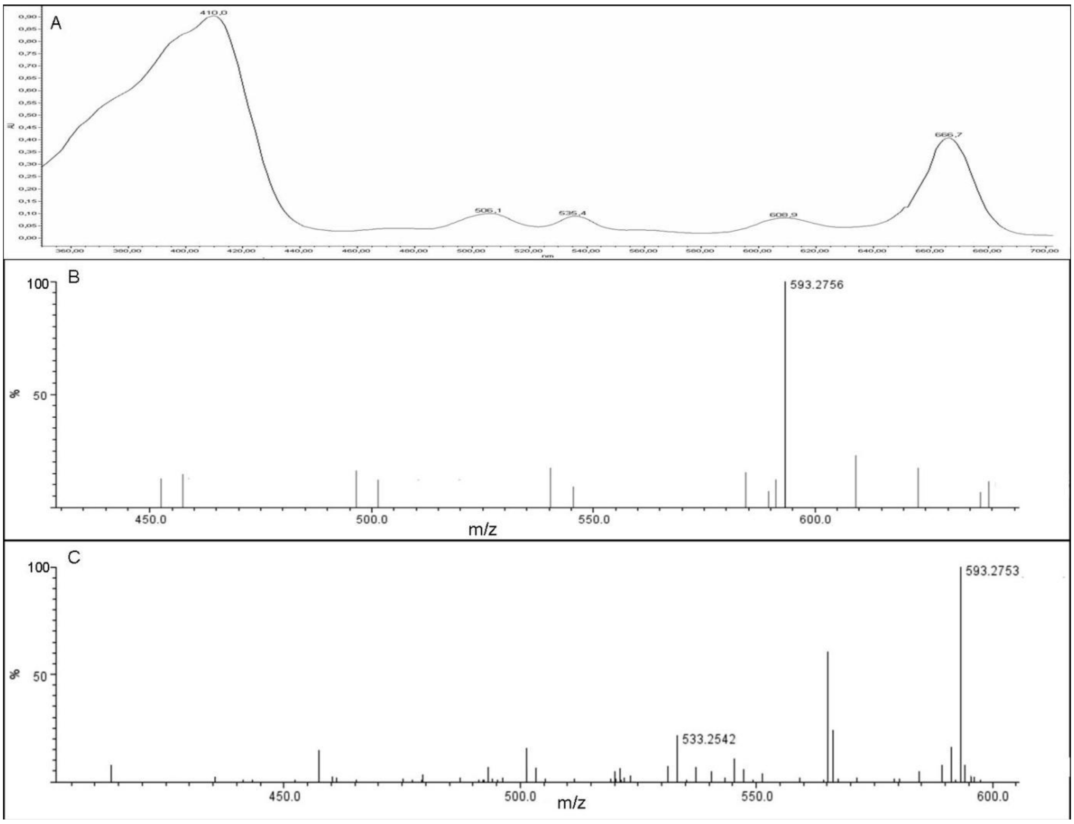

Figure S6. UV-vis (**A**), high resolution MS (**B**) and MSMS (**C**) spectra of standard pyropheophorbide a.

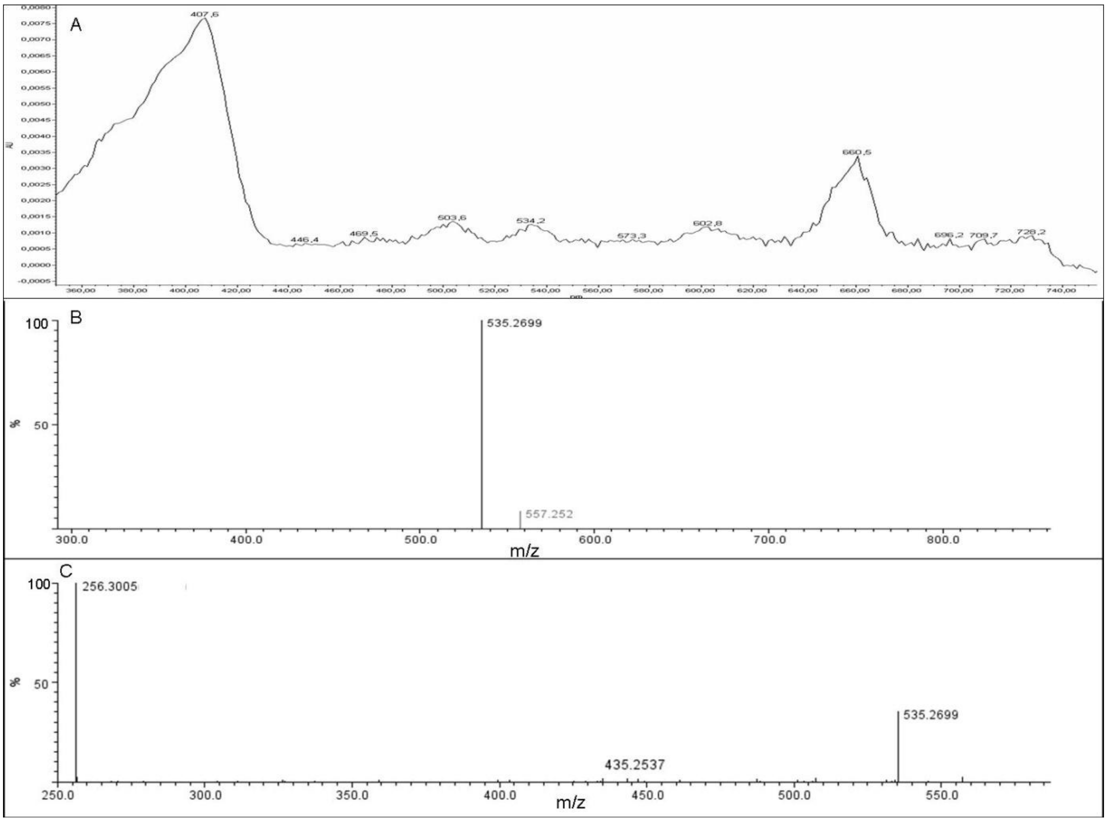

Figure S7. UV-vis (**A**), high resolution MS (**B**) and MSMS (**C**) spectra of standard zeaxanthin.

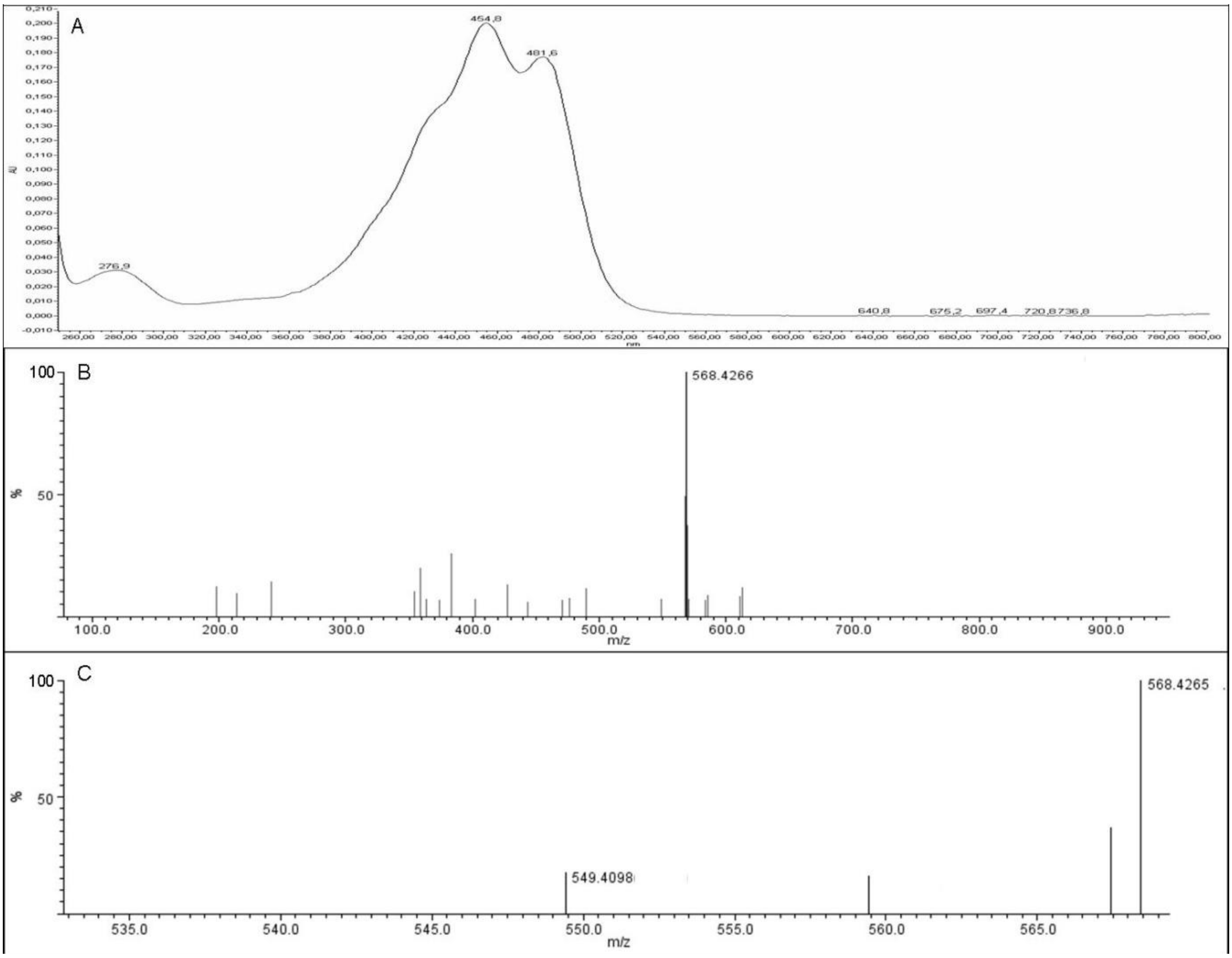

Figure S8. UV-vis (**A** and **D**), high resolution MS (**B** and **E**) and MSMS (**C** and **F**) spectra of Fraction 4. The **A**, **B** and **C** spectra correspond to pyropheophorbide a. The **D**, **E** and **F** spectra correspond to zeaxanthin.

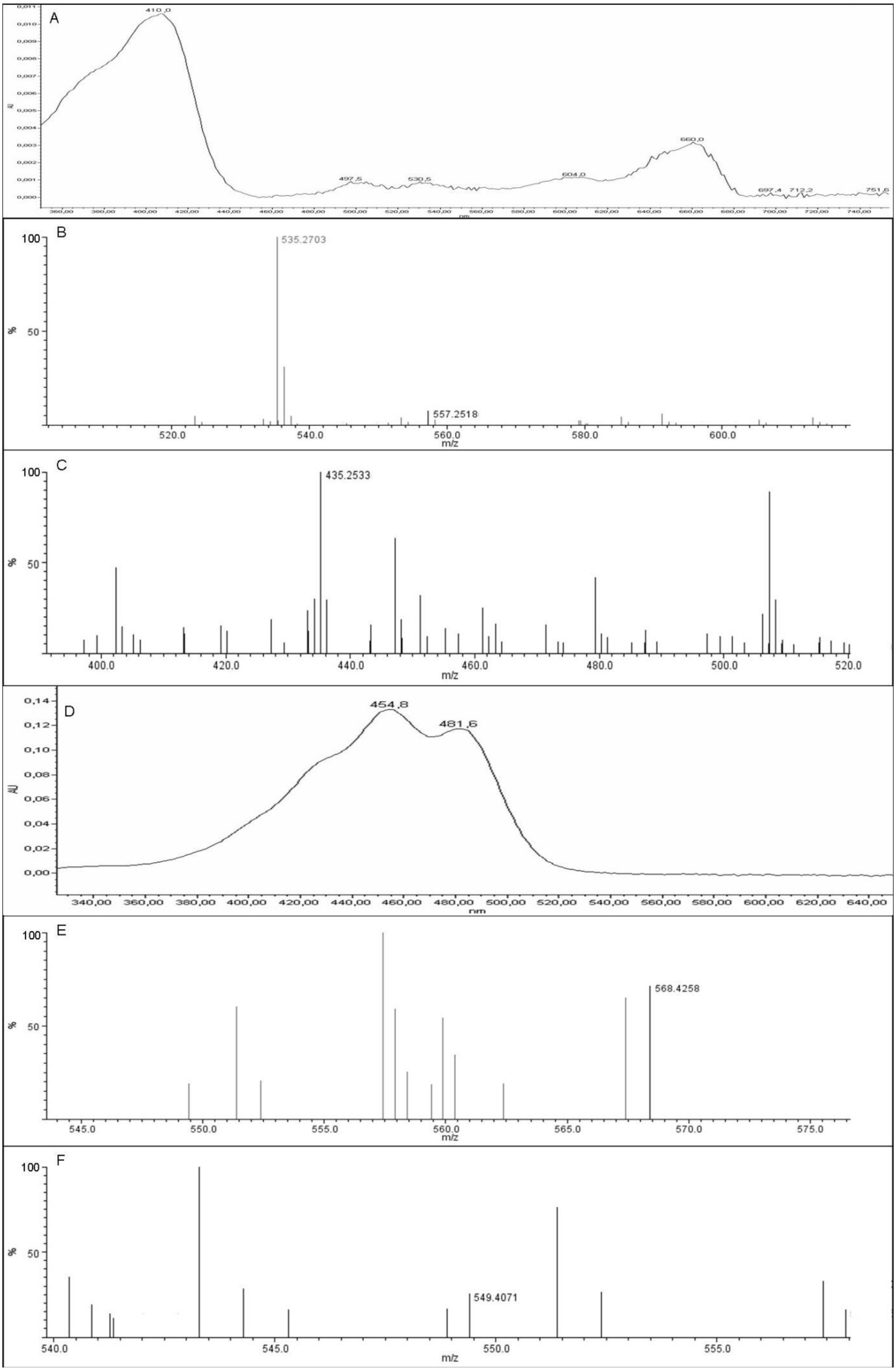

Figure S9. UV-vis (**A**), high resolution MS (**B**) and MSMS (**C**) spectra of standard β-cryptoxanthin.

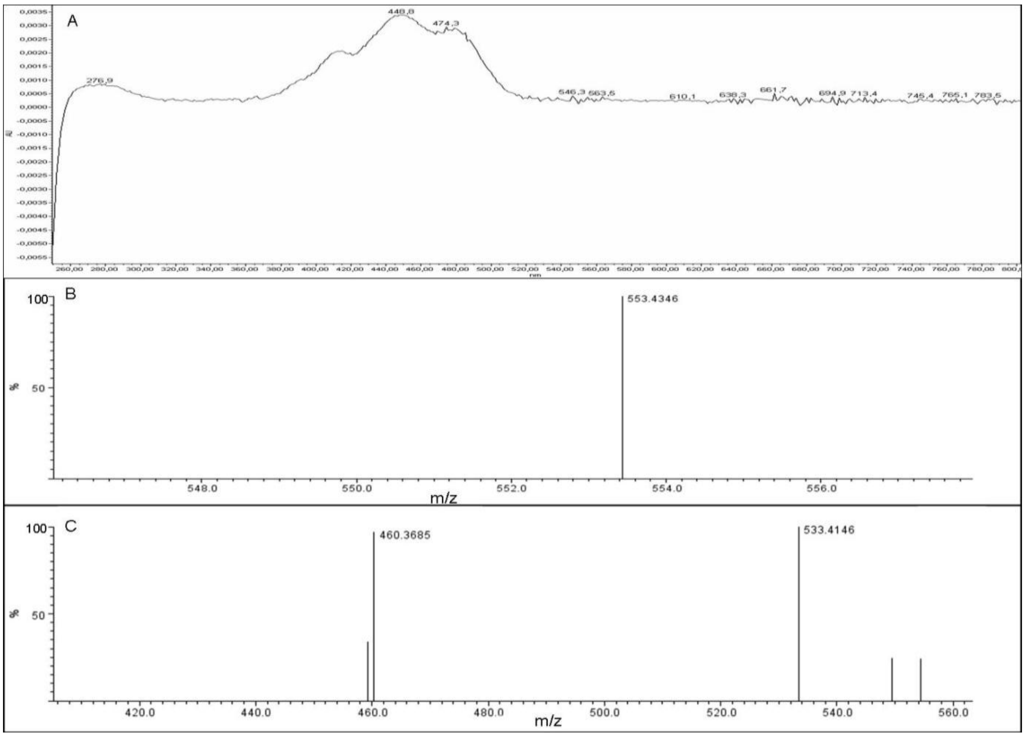

Figure S10. UV-vis (**A** and **B**), high resolution MS (**C**) and MSMS (**D**) spectra of Fraction 5. The **A** spectrum corresponds to 5,6-epoxy-β-cryptoxanthin. The **B**, **C** and **D** spectra correspond to β-cryptoxanthin.

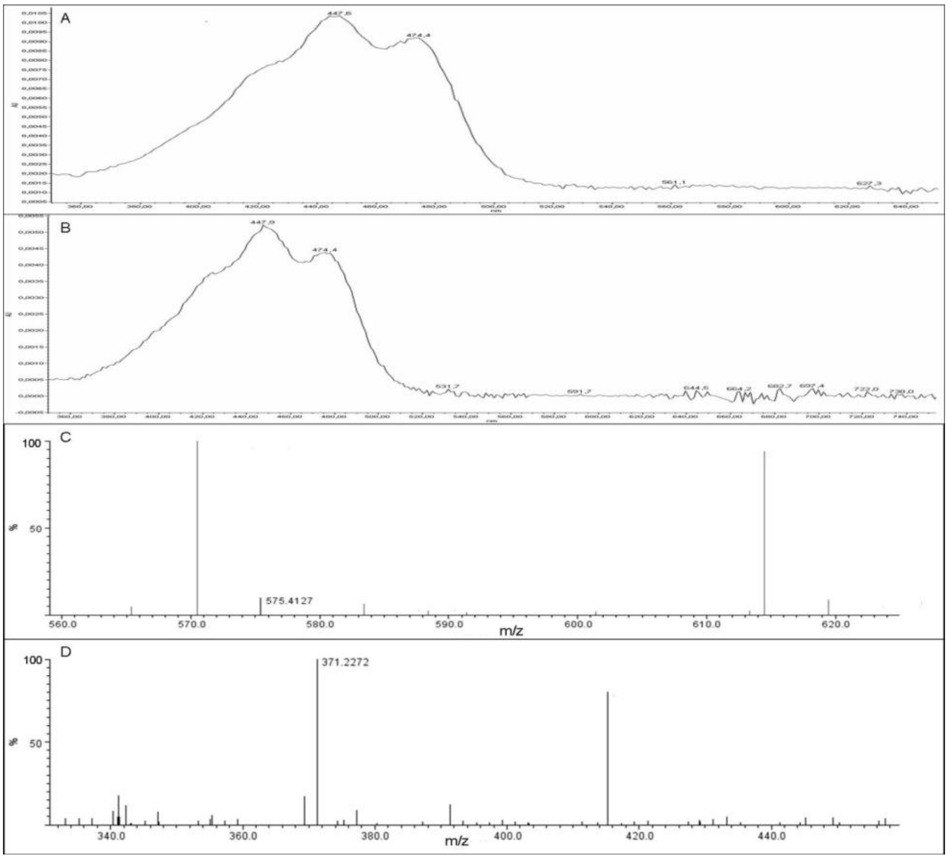

Figure S11. UV-vis (**A**), high resolution MS (**B**) and MSMS (**C**) spectra of standard chlorophyll a.

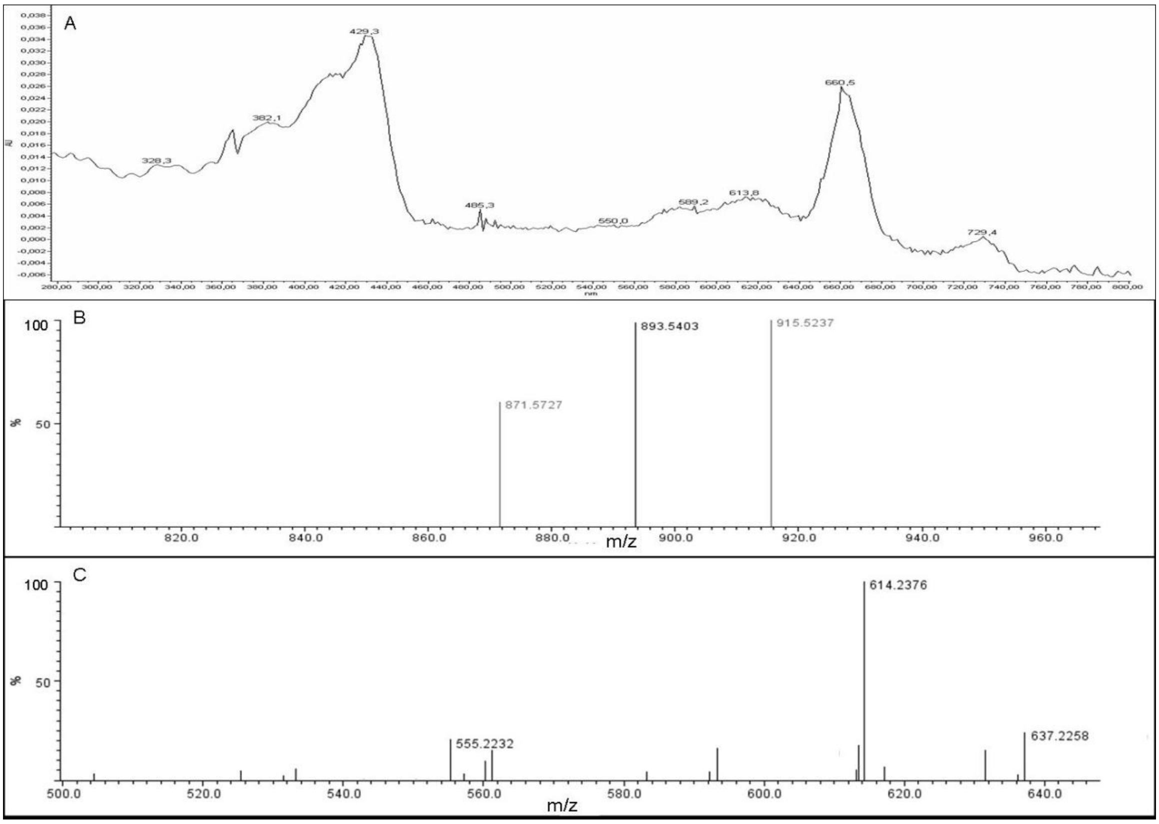

Figure S12. UV-vis (**A**, **D** and **G**), high resolution MS (**B** and **E**) and MSMS (**C** and **F**) spectra of Fraction 6. The **A**, **B** and **C** spectra correspond to chlorophyll a. The **D**, **E** and **F** spectra correspond to pheophytin a. The **G** spectrum corresponds to the unidentified carotenoid.

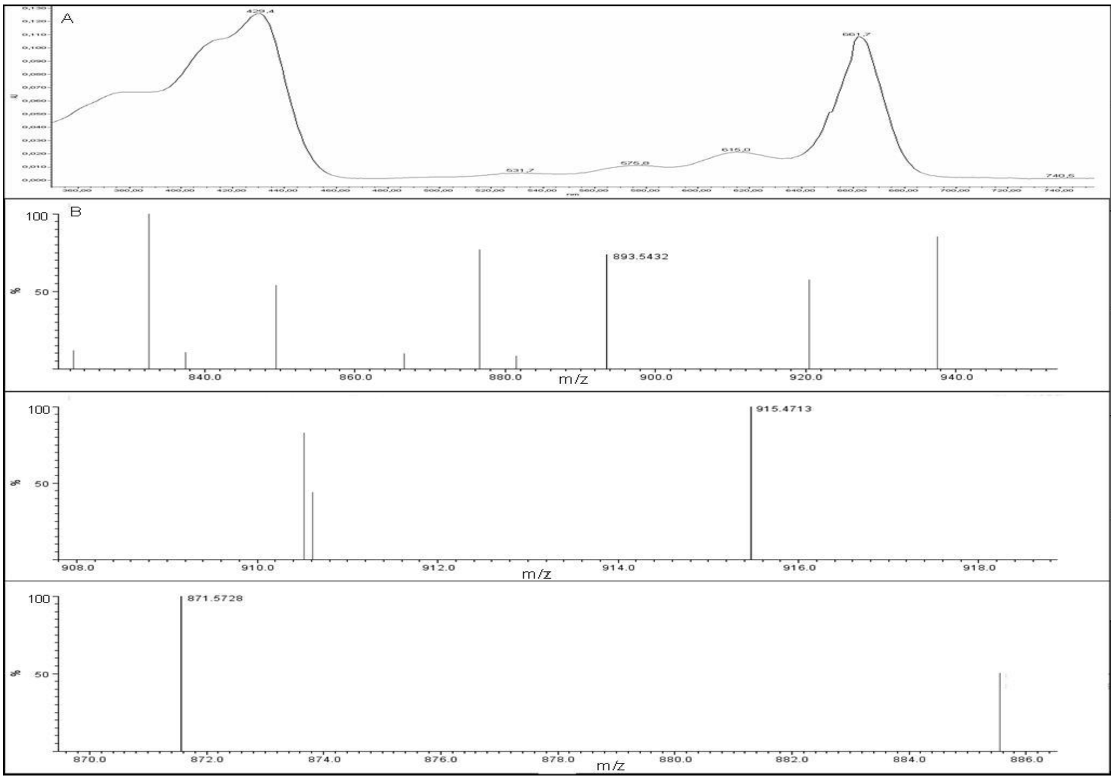

Figure S12. *Cont.*

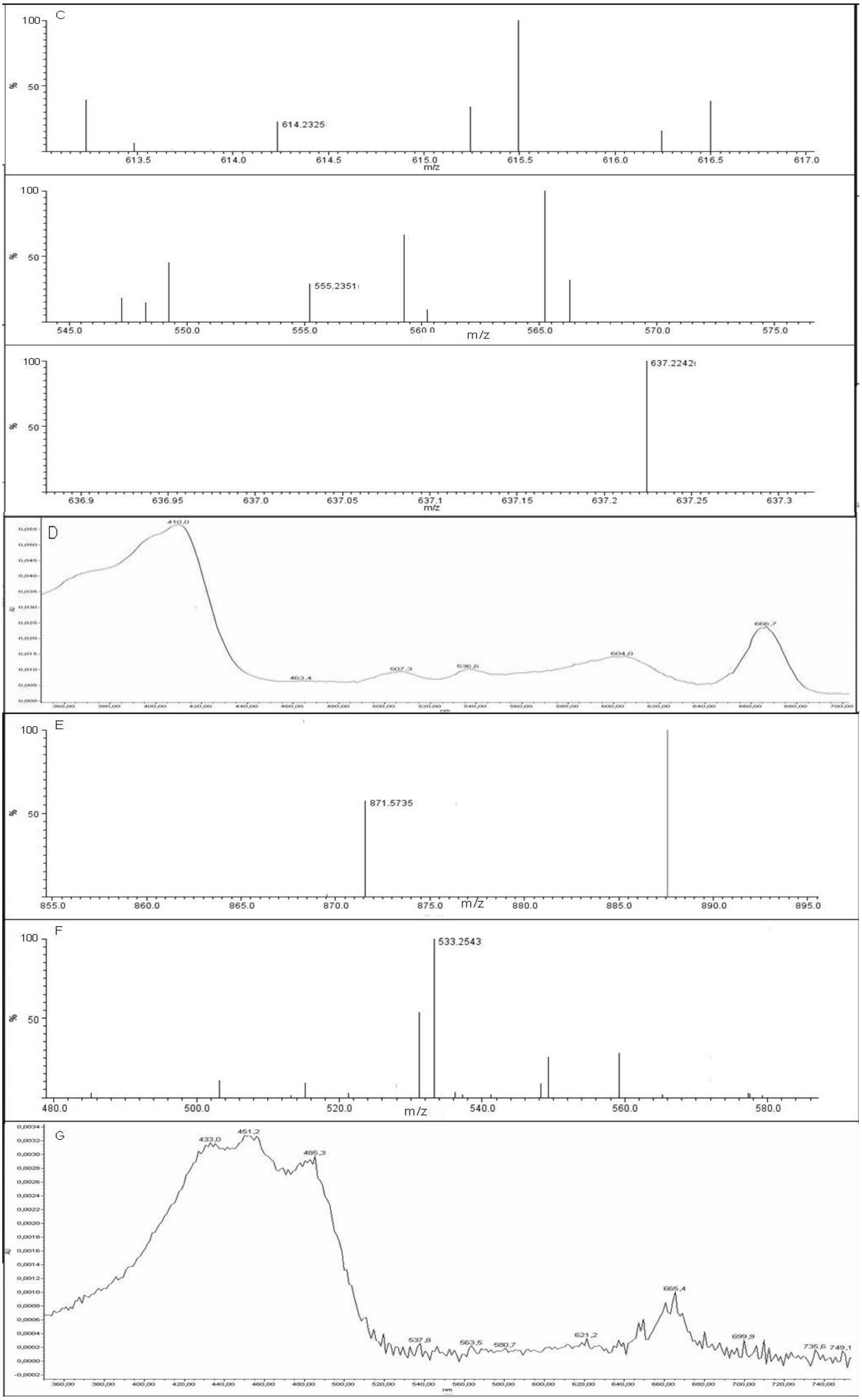

Figure S13. UV-vis (**A**, **D**, **G**, **H**, **I** and **J**), high resolution MS (**B** and **E**) and MSMS (**C** and **F**) spectra of Fraction 7. The **A**, **B** and **C** spectra correspond to chlrorophyll a epimer. The **D**, **E** and **F** spectra correspond to pheophytin a. The **G** spectrum corresponds to DV-pheophytin a. The **H** spectrum corresponds to MV-pheophytin a. The **I** spectrum corresponds to mutatochrome. The J spectrum corresponds to purpurin-7-phytyl ester.

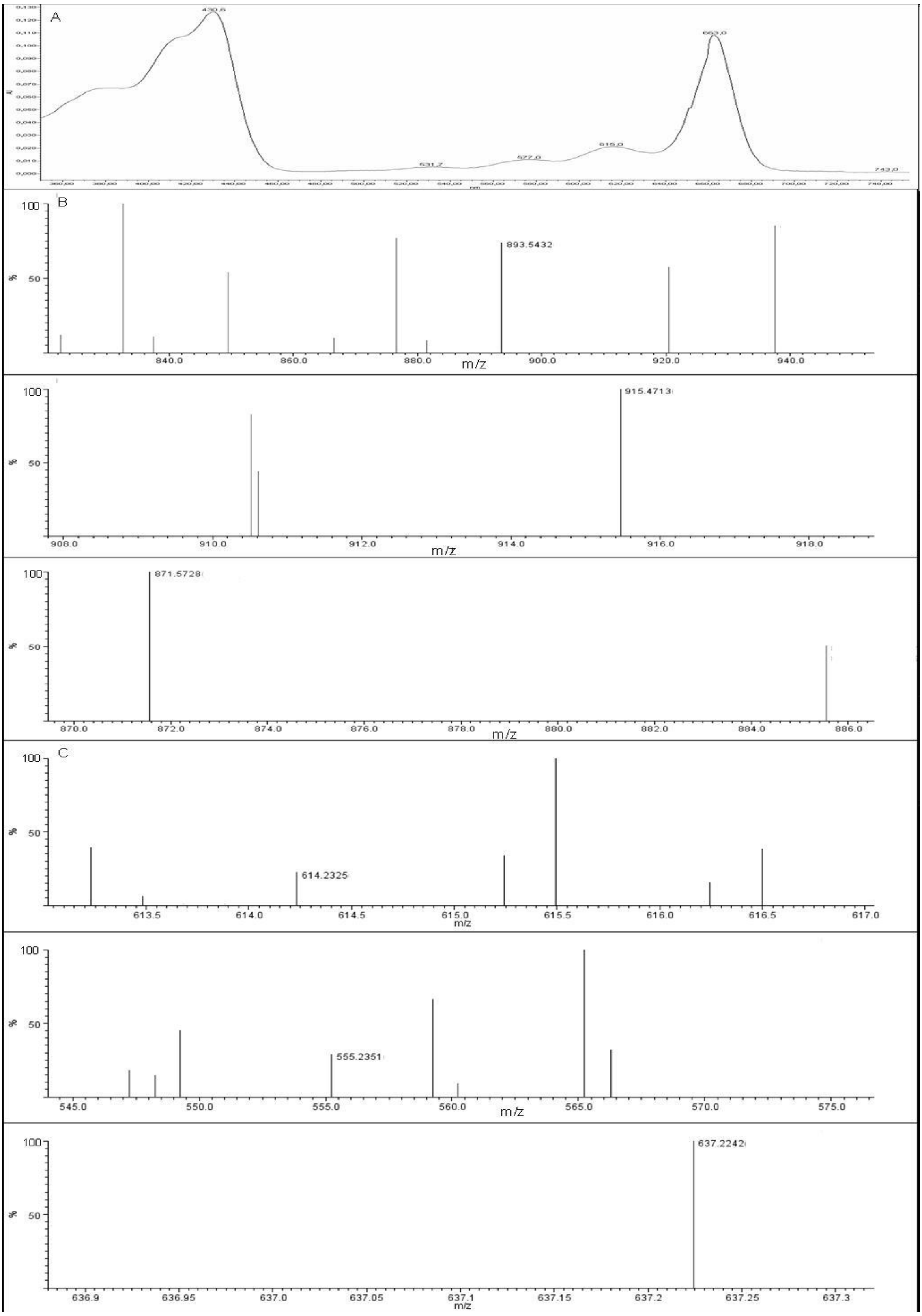

Figure S13. *Cont.*

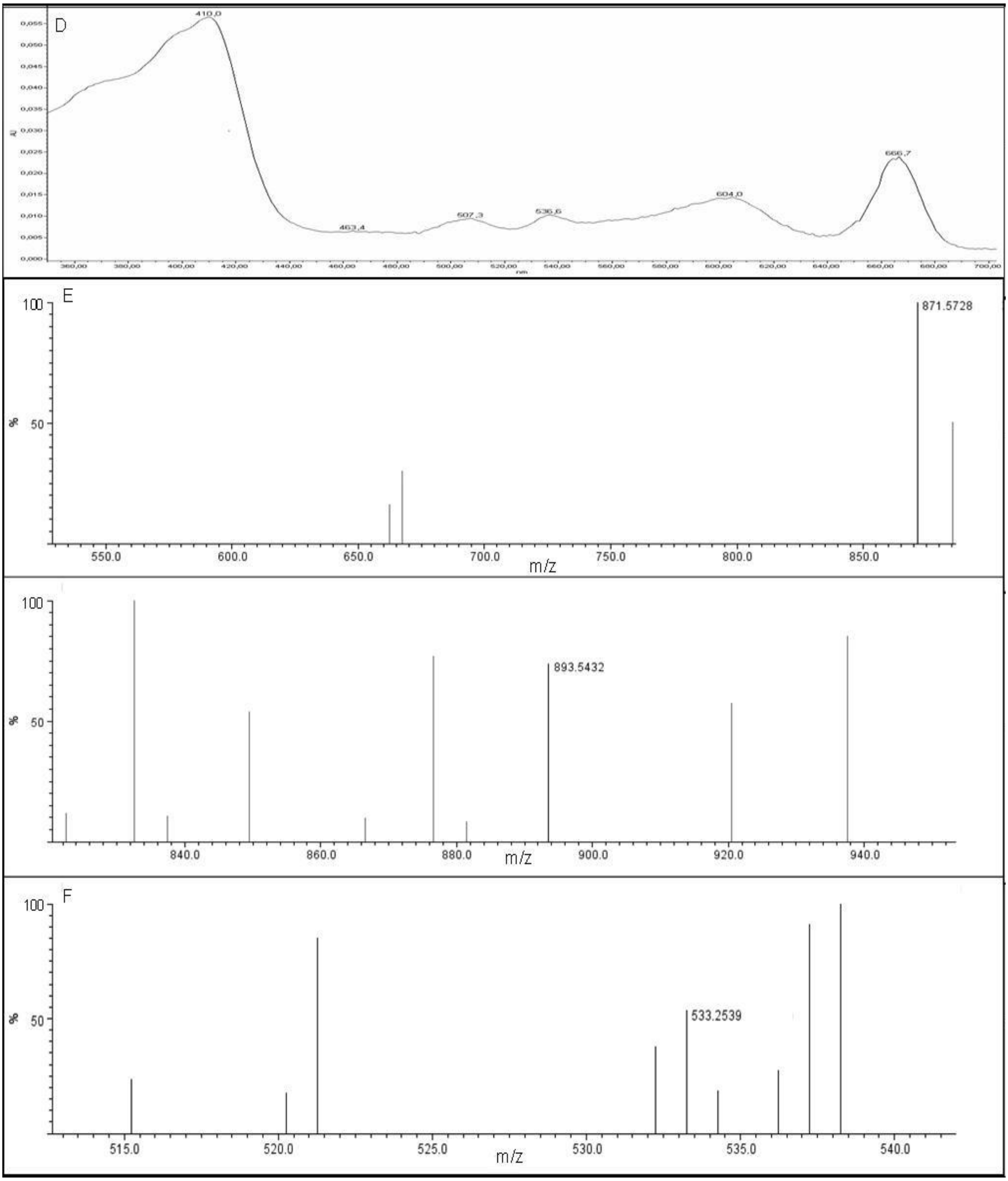

Figure S13. *Cont.*

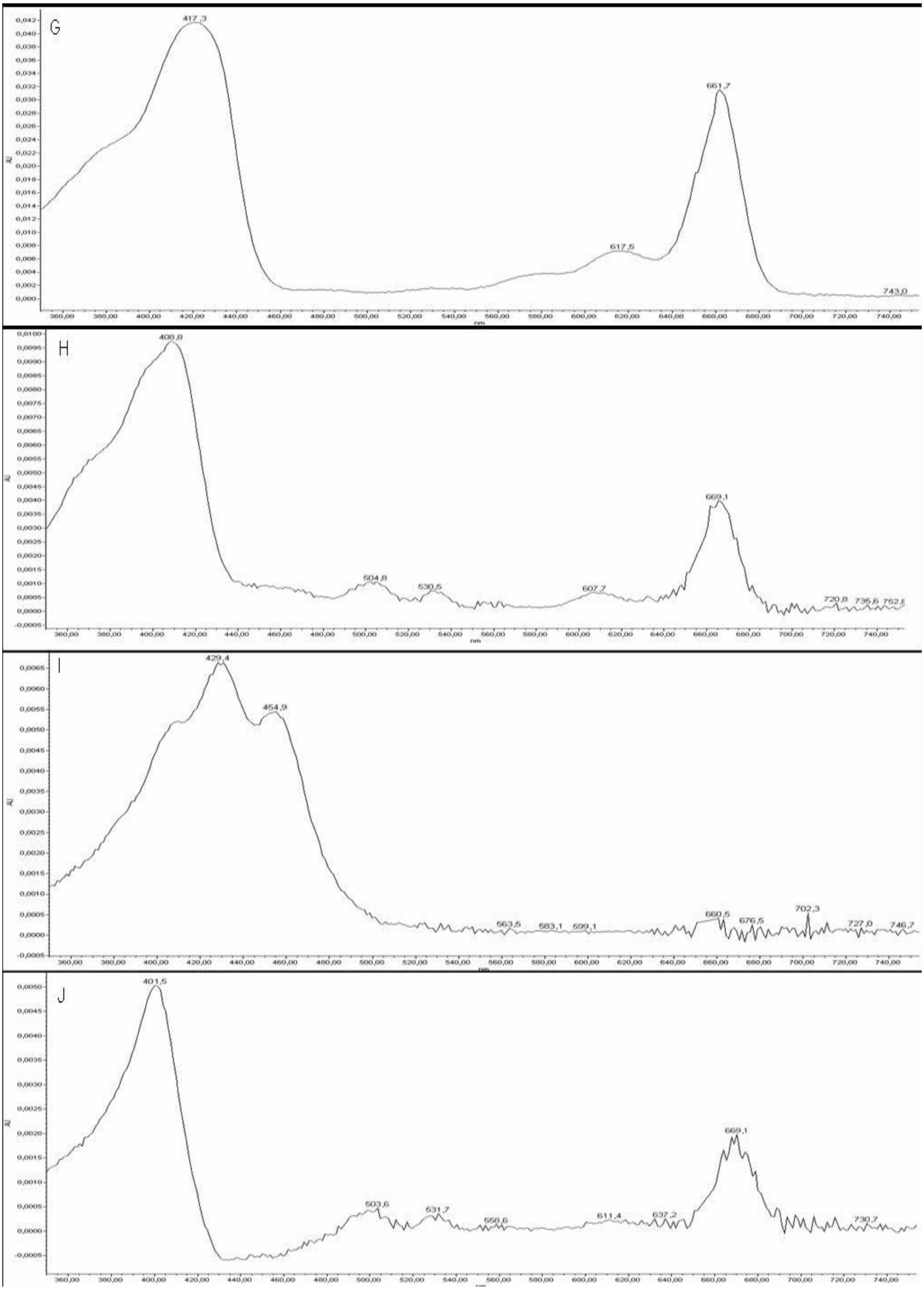

Figure S14. UV-vis (**A**), high resolution MS (**B**) and MSMS (**C**) spectra of standard β-Carotene.

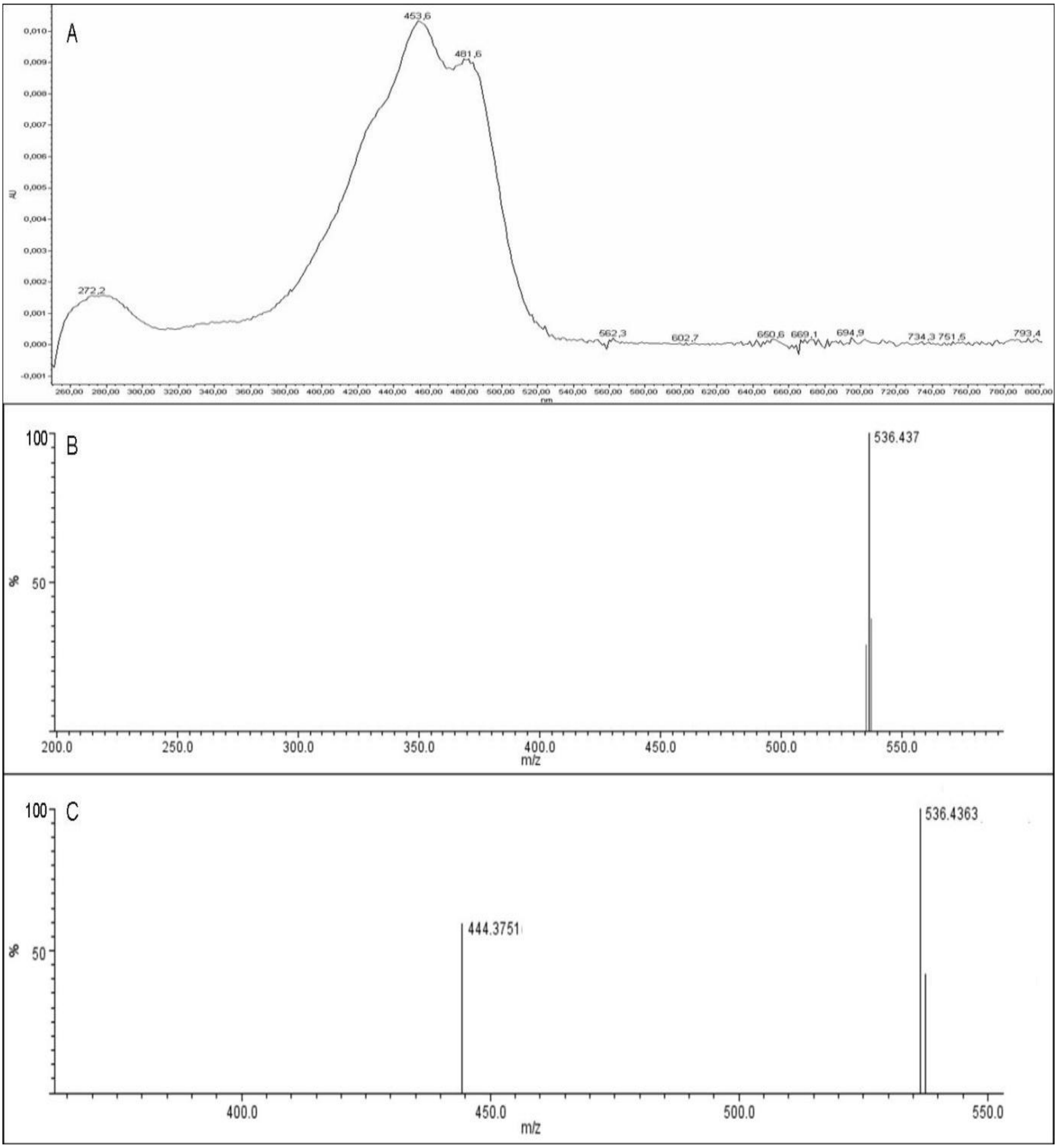

Figure S15. UV-vis (**A**) and high resolution MS (**B**) spectra of Fraction 8 (β-carotene).

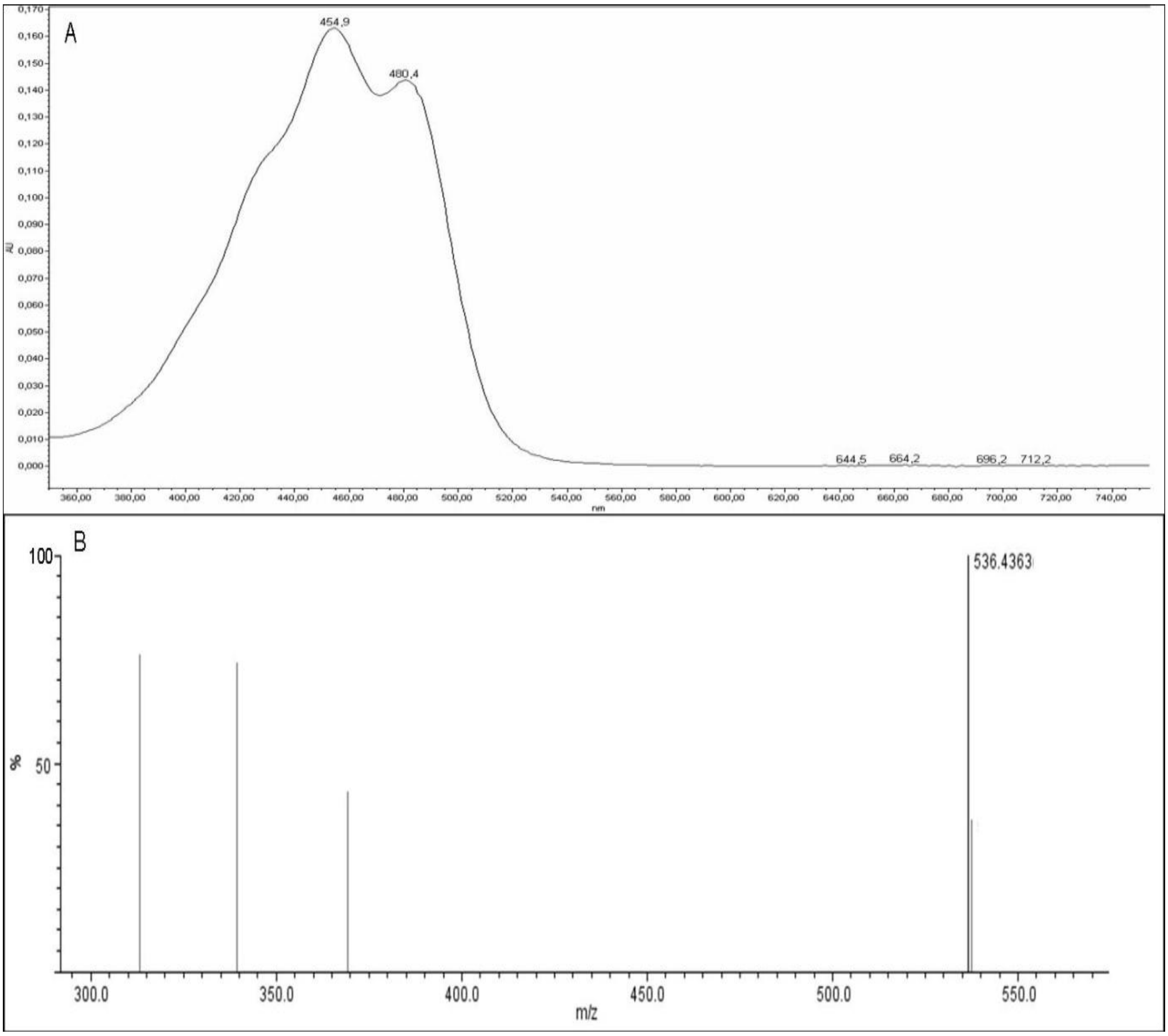

© 2013 by the authors; licensee MDPI, Basel, Switzerland. This article is an open access article distributed under the terms and conditions of the Creative Commons Attribution license (http://creativecommons.org/licenses/by/3.0/).
